# The kinesin of the flagellum attachment zone in *Leishmania* is required for cell morphogenesis, cell division and virulence in the mammalian host

**DOI:** 10.1371/journal.ppat.1009666

**Published:** 2021-06-18

**Authors:** Rosa Milagros Corrales, Slavica Vaselek, Rachel Neish, Laurence Berry, Camille D. Brunet, Lucien Crobu, Nada Kuk, Julio Mateos-Langerak, Derrick R. Robinson, Petr Volf, Jeremy C. Mottram, Yvon Sterkers, Patrick Bastien

**Affiliations:** 1 Research Unit “MiVEGEC”, University of Montpellier, CNRS, IRD, Academic Hospital (CHU) of Montpellier, Montpellier, France; 2 Department of Parasitology, Charles University, Prague, Czech Republic; 3 York Biomedical Research Institute and Department of Biology, University of York, York, United Kingdom; 4 Research Unit “LPHI” (Laboratory of Pathogen Host Interactions), University of Montpellier, CNRS, Montpellier, France; 5 Institut de Génétique Humaine, University of Montpellier, CNRS, Montpellier, France; 6 Research Unit “Fundamental Microbiology and Pathogenicity”, “Protist Parasite Cytoskeleton (ProParaCyto)”, University of Bordeaux, UMR 5234, CNRS, Bordeaux, France; University of California, Los Angeles, UNITED STATES

## Abstract

*Leishmania* parasites possess a unique and complex cytoskeletal structure termed flagellum attachment zone (FAZ) connecting the base of the flagellum to one side of the flagellar pocket (FP), an invagination of the cell body membrane and the sole site for endocytosis and exocytosis. This structure is involved in FP architecture and cell morphogenesis, but its precise role and molecular composition remain enigmatic. Here, we characterized *Leishmania* FAZ7, the only known FAZ protein containing a kinesin motor domain, and part of a clade of trypanosomatid-specific kinesins with unknown functions. The two paralogs of FAZ7, FAZ7A and FAZ7B, display different localizations and functions. FAZ7A localizes at the basal body, while FAZ7B localizes at the distal part of the FP, where the FAZ structure is present in *Leishmania*. While null mutants of FAZ7A displayed normal growth rates, the deletion of FAZ7B impaired cell growth in both promastigotes and amastigotes of *Leishmania*. The kinesin activity is crucial for its function. Deletion of FAZ7B resulted in altered cell division, cell morphogenesis (including flagellum length), and FP structure and function. Furthermore, knocking out FAZ7B induced a mis-localization of two of the FAZ proteins, and disrupted the molecular organization of the FP collar, affecting the localization of its components. Loss of the kinesin FAZ7B has important consequences in the insect vector and mammalian host by reducing proliferation in the sand fly and pathogenicity in mice. Our findings reveal the pivotal role of the only FAZ kinesin as part of the factors important for a successful life cycle of *Leishmania*.

## Author summary

*Leishmania* are flagellated trypanosomatid parasites causing worldwide human and animal diseases. As ’divergent eukaryotes’, their biology presents unique features and structures, of which the specific functions constitute potential drug targets. Among others, they possess a unique cytoskeletal structure termed the flagellum attachment zone (FAZ) attaching the base of their flagellum to one side of the flagellar pocket (FP), which is the sole site for endocytosis and exocytosis. The FP together with other unique flagellum-associated structures are crucial for parasite survival, but the functioning of this whole remains largely enigmatic. *Leishmania* also possess an expanded repertoire of kinesins (>55), including two trypanosomatid-specific families. Here, we show that the deletion of the sole kinesin among FAZ proteins disrupts cell morphogenesis, FP organisation and cell division. Furthermore, the ability to proliferate in the insect vector and mammalian host is reduced in parasites lacking the kinesin FAZ7B. This study helps elucidate the factors contributing to the successful lifecycle and pathogenicity of the parasite. It also highlights the functional diversification of motor proteins during evolution.

## Introduction

**Fig 1:**
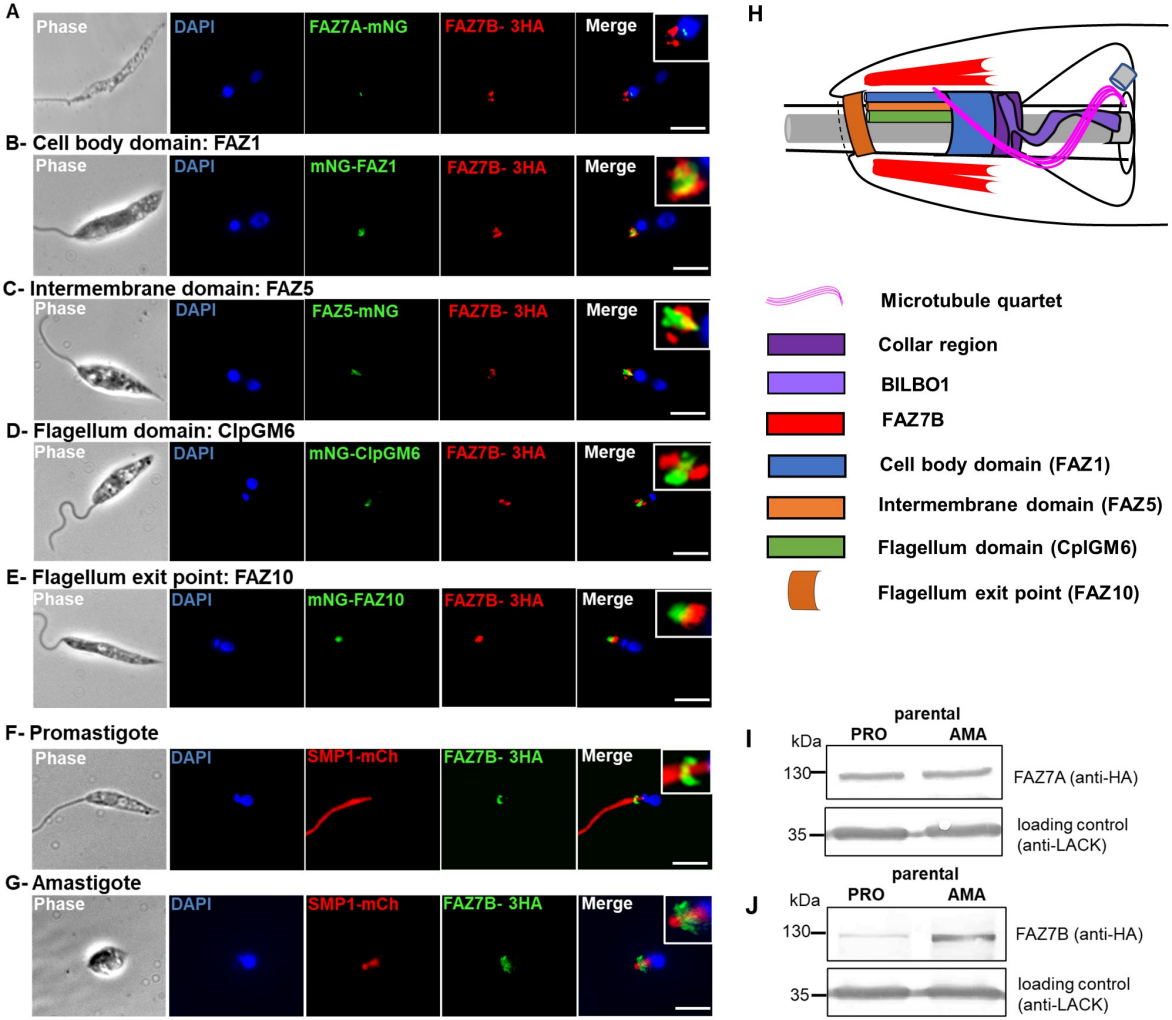
*L*. *mexicana* FAZ7B localizes at the cell body side of the FAZ and is differentially expressed in promastigote and amastigote forms. Immunofluorescence assay were performed as indicated in Materials and Methods. (A) Extracted cytoskeleton of a *L*. *mexicana* promastigote expressing FAZ7A-mNG (green) and FAZ7B-3xHA (red). (B-E) Whole promastigote cells of *L*. *mexicana* expressing FAZ7B-3xHA (red) and the indicated fusion proteins of FAZ components (green). (F-G) Whole promastigote (F) and axenic amastigote (G) cells from *L*. *mexicana* expressing FAZ7B-3xHA (green) and the flagellar membrane marker SMP1-mCh (red). DNA was labelled with DAPI (blue). Scale bar: 5μm. (H) Schematic of the FAZ protein localisations and anterior cell tip organisation in a promastigote cell, providing a side-on view of the FAZ (as inferred from our data and adapted from Halliday et al., PLoS Pathog 16(10): e1008494). The flagellar pocket is divided into two regions (bulbous and neck) joining by the flagellar pocket collar region; the microtubule quartet (MTQ) starts at the basal bodies and wraps around the bulbous region of the flagellar pocket before terminating in the neck region. The different FAZ domains and the proteins analysed in the present study are shown. Not to scale. (I-J) Western blot of promastigotes (PRO) and axenic amastigotes (AMA) of the parental cell line expressing FAZ7A-3xHA (I) and FAZ7B-3xHA (J); 35 μg of total protein extract were loaded on a 12% SDS-PAGE, transferred and immuno-probed with anti-HA and anti-LACK (loading control) antibodies.

*Leishmania spp*. are trypanosomatid parasites responsible for leishmaniasis, a major human and animal neglected disease present in 98 countries in four continents. *Leishmania* has a digenetic life cycle that alternates between an insect vector and a mammalian host. Within the sand fly vector, *Leishmania* is an extracellular parasite, termed promastigote, characterized by a long and motile flagellum exiting at the anterior end of an elongated cell body. Within the mammalian host, *Leishmania* is an obligate intracellular parasite, termed amastigote, with a rounded cell body and a short non-motile flagellum. In both stages, the external cytoskeleton is made of a helical, rigid, highly organised subpellicular ’corset’ of interlinked microtubules running parallel. Differentiation from the promastigote to the amastigote stage involves a drastic remodelling of this sub-pellicular microtubule array and the restructuring of the flagellum from a motile 9+2 axoneme to a collapsed 9+0 (9v) organization [1]. At the base of the flagellum is an invagination of the cell membrane called the flagellar pocket (FP), which is the only site for endocytosis and exocytosis. The FP has key roles in several cellular processes, including flagellum assembly, cell shape, cell division and immune evasion [2] hence is crucial for parasite pathogenicity. The FP comprises two regions: the bulbous lumen, immediately anterior/distal to the basal body, and the distal FP neck [3] (see schematic in Fig 1H). At the neck, the FP and flagellum membranes are closely apposed until the flagellum emerges from the cell [4]. Between the bulbous lumen and the neck is the flagellar pocket collar (FPC), a cytoskeletal structure essential for biogenesis and function of the FP [5,6]. A set of specific microtubules is associated with the flagellar pocket. Four specialised parallel microtubules that form the microtubule quartet (MTQ) run as a short tight array along the FP surface and terminate irregularly in the FP neck region [4]. Two *Leishmania*-specific sets of microtubules, termed pocket microtubules and cytoplasmic microtubules, are nucleated from the FPC area and run along the bulbous region of the FP and through the cytoplasm, respectively [4].

The flagellum attachment zone (FAZ) is a complex cytoskeletal structure connecting the cell body cytoskeleton to the flagellar cytoskeleton across both the flagellum and cell body membranes [7]. It has been extensively studied in the related parasite *Trypanosoma brucei*, where it runs with the flagellum from the posterior end all along the cell body [7]. In *Leishmania*, the FAZ is a discrete structure associated with the neck of the FP, anchoring the base of the flagellum to one side of the FP neck region. It includes a (FAZ) filament and electron-dense attachment areas comprising irregularly spaced junctional complexes [4]. Its precise role and molecular architecture remain largely enigmatic. Intriguingly, in *Leishmania*, unlike *T*. *brucei*, there is a spatial separation between the junctional complexes and the FAZ filament [4]. In both parasites, the biogenesis of the FP and FAZ are linked; and a physical connection between the FAZ and FP cytoskeleton has been shown in *T*. *brucei*, mediated by the MTQ [3,8] and the bilobe structure [9].

A large number (≥34) of genes encoding FAZ proteins in *T*. *brucei* has been published over the years (reviewed in [7], and their orthologs are present in the *Leishmania* genome. Depletion of FAZ components in *T*. *brucei* has shown that it is a key morphogenetic structure regulating flagellum axial positioning, cell morphology, single-copy organelle positioning and cytokinesis [10–14]. Deciphering the role of FAZ components in *Leishmania* is thus important for understanding the interplay between the FAZ and the FP function that likely orchestrates cell morphology throughout its life cycle [4]. To date, only two FAZ proteins, FAZ5 and FAZ2, have been characterized in *Leishmania*, and they play a role in FP architecture and morphogenesis of the cell anterior tip [15,16]. Here we focussed on FAZ7 which is the only FAZ protein which contains a kinesin motor domain. Kinesins are ubiquitous motor proteins whose most common function is transporting cargos along microtubules. This super-family comprises 19 clades [17–19]; of these, two families are trypanosomatid-specific, and FAZ7 belongs to the "Tryp2" specific family. In *T*. *brucei*, FAZ7 localizes at the distal end of the FAZ filament, where the flagellum separates from the cell body; its function remains unknown [20]. In the *Leishmania* genome, the FAZ7 ortholog is duplicated; thus, we termed the paralogs FAZ7A and FAZ7B. Here, we show that FAZ7A localizes at the basal body while FAZ7B localizes at the anterior/distal part of the FP, where the FAZ structure is present in *Leishmania*. While null mutants of the basal body FAZ7A display normal growth rates, the deletion of FAZ7B alters several essential cellular processes. This has important consequences by reducing proliferation in the insect vector and the mammalian host.

## Results

### *Leishmania mexicana* FAZ7B localizes at the cell body side of the FAZ and is differentially expressed in promastigote and amastigote stages

Two paralogs of *T*. *brucei* FAZ7 (Tb10.15390) are encoded in the *L*. *mexicana* genome (https://tritrypdb.org/tritrypdb/), that we termed FAZ7A (LmxM.19.0680) and FAZ7B (LmxM.19.0690). Sequence alignment of *T*. *brucei* FAZ7 (TbFAZ7) with these two paralogs, excluding the kinesin motor domain, revealed a low protein identity (~25%). Sequence alignment of the full sequence of *L*. *mexicana* (Lmx) FAZ7A and FAZ7B showed a well conserved kinesin motor domain with 52% amino acid identity, but only 28% protein identity without the motor domain sequence (S1 Fig). In both proteins, the motor domain is located near the N-terminal end; and both paralogs belong to the trypanosomatid-specific “Tryp2” kinesin family, of unknown function [18,19].

To determine the subcellular localization of the two paralogs in *L*. *mexicana*, we generated promastigote parasites expressing proteins tagged at their endogenous loci using the CRISPR-Cas9 PCR-based strategy [21]. All FAZ7 protein-tagging experiments subsequently performed in this study were done in the same way. FAZ7A was tagged with a 3xHA epitope or mNeonGreen (mNG); and FAZ7B with a 3xHA epitope or m-Cherry (mCh), all at the C-terminal end. The signal observed for each protein was identical whichever the nature of the tag. Immunofluorescence imaging revealed a different localization of the two FAZ7 paralogs. FAZ7A localized at the pro-basal and basal body (Fig 1A), as shown by colocalization with the mCh-tagged basal body protein Centrin4 (S2A Fig) [22]. By contrast, FAZ7B was present as a relatively heterogeneous signal at the anterior end of the cell body (Fig 1), consistent with a localization at the FAZ [4]. The fluorescence signal of both proteins remained associated with extracted cytoskeletons, suggesting that both are cytoskeletal proteins (Fig 1A). To better define this localization, we tagged the SMP1 flagellum membrane marker [23] with mCh to map the FP organization [4] and performed cell imaging at different planes in the z-axis. FAZ7B displayed a clearly asymmetric ring structure around the distal part of the FP area, the ring being thicker and longer on one side of the flagellum, and much thinner and sometimes hardly visible on the other side (Fig 1F and S1 Movie). We then examined the respective localization of FAZ7B and representative components of the FAZ. FAZ7B localized in close proximity with FAZ1, FAZ2, FAZ8, as well as FAZ5 (Fig 1B and 1C, S2B and S2C Fig). FAZ1, FAZ2, and FAZ8 label the cell body side of the FAZ, while FAZ5 is a component of the ’cell body membrane domain’ of the FAZ [4,15,16]. In z-stack images, the FAZ7B signal appeared to be slightly proximal and around or on one side of that of FAZ1 (S2 Movie) which formed a ‘horse-shoe’ around the flagellar pocket. 3D-SIM super-resolution in whole cell and cytoskeleton confirmed these observations, showing FAZ7B surrounding a ring formed by FAZ1, and revealed that there was no colocalization between both proteins (S3 and S4 Movies, and S2D and S2E Fig). FAZ5 is localized along the entire length of the cytoplasmic side of the FAZ in the pocket neck [4,15]. In z-stack images, FAZ7B was also located around FAZ5, rarely proximal to it. The FAZ7B signal was also around and slightly proximal to ClpGM6 (Fig 1D), representative of the ’flagellum’ side of the FAZ [4,15,16]. Finally, FAZ7B was always proximal and often contiguous to FAZ10, a marker of the flagellum exit point (Fig 1E) [4]. The whole of these data is summarized in a cartoon in Fig 1H. Taken together, our data strongly suggest that FAZ7B is localized at the cell side of the FAZ.

We next compared the localization and expression of FAZ7B in the promastigote and axenic amastigote stages. To this end, we tagged both alleles of FAZ7B gene with a 3xHA tag at the C-terminus and confirmed the correct integration of the tag and the absence of any FAZ7B wild-type allele by PCR (S3A Fig). In amastigotes, the constriction in flagellum width in the FP neck [4] was visible in the SMP1-mCh labelling; and FAZ7B showed a larger signal spreading across and distally from the FP neck constriction (Fig 1G). The protein levels of HA-tagged FAZ7 were quantified in promastigote and axenic amastigotes using the scaffold protein LACK (Leishmania’s receptor for activated C-kinase) [24] as a control for normalization. Densitometric analysis of the bands showed that the protein level of FAZ7B was increased four-fold in the axenic amastigote form (Fig 1J) while no differences were detected for FAZ7A (Fig 1I). These data suggest a differential expression of FAZ7B between the promastigote and amastigote stages.

### FAZ7B, but not FAZ7A, is required for normal cell growth in promastigote and amastigote stages

**Fig 2:**
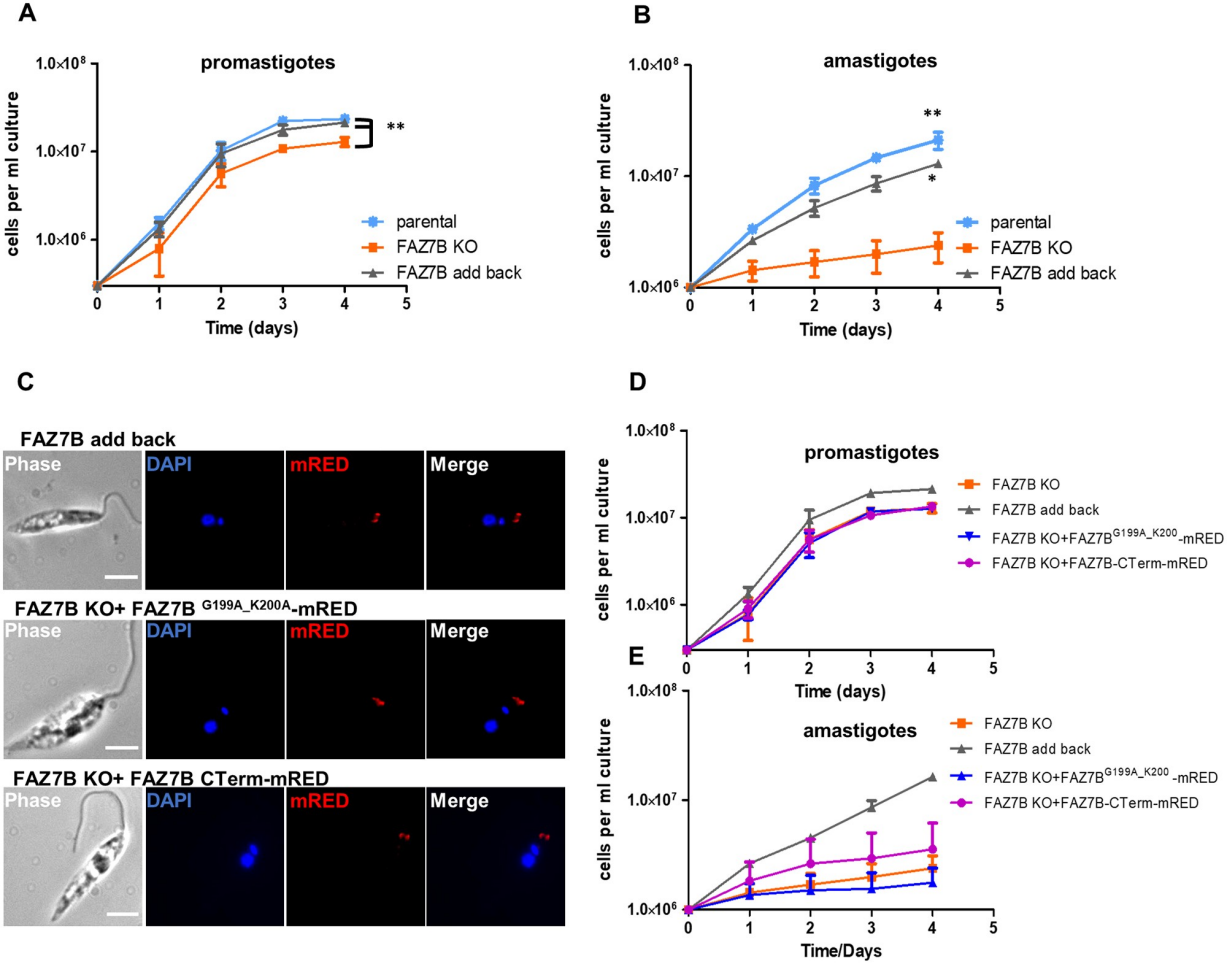
FAZ7B is required for normal cell growth in promastigote and amastigote stages. (A-B) Representative growth curves (log scale) of promastigotes (A) and axenic amastigotes (B) of the parental, FAZ7B KO and FAZ7B add-back cell lines over a 4-day course. Cell density was determined by counting at 24 h intervals and the mean ± SD of three independent experiments was plotted. *p < 0.05, **p < 0.01 (Student’s *t*-test). (C) Fluorescence micrographs of whole cells from FAZ7B add-back (upper panel) and mutant-complemented cell lines: FAZ7B KO + FAZ7B ^G199A_K200A^-mRED (middle panel) and FAZ7B KO + FAZ7B C-term-mRED (lower panel) cell lines. Scale bar: 5μm. Growth curves of promastigotes (D) and axenic amastigotes (E) of the FAZ7B KO, FAZ7B add-back, FAZ7B KO+ FAZ7B ^G199A_K200A^-mRED and FAZ7B KO+ FAZ7B C-term-mRED cell lines over a 4-day course. Cell density was determined by counting at 24 h intervals and mean ± SD of triplicate values was plotted.

To understand the role of *L*. *mexicana* FAZ7, we generated null mutants of FAZ7A, FAZ7B and a double knock-out (KO) FAZ7A+B. Gene deletion mutants were generated by replacement of both alleles of the FAZ7 CDSs with antibiotic resistance genes in a parental cell line expressing CAS9 for generation of DNA double-strand break and the T7 RNA polymerase for *in vivo* transcription of guide RNA [21]. All deletions were confirmed by PCR (S3B–S3D Fig). The growth of promastigotes and axenic amastigotes of these null mutants was assessed by counting the cells at different time points over four days. Null mutants of the basal body-resident FAZ7A exhibited a normal growth rate in both promastigote and amastigote stages compared to the parental cell line (S4A and S4B Fig). By contrast, deletion of FAZ7B and the FAZ7A+B double KO impaired cell growth in both promastigotes and axenic amastigotes, but much more drastically in the amastigote stage which showed a strong growth arrest (Fig 2A and 2B, S4A and S4B Fig).

Immunofluorescence labelling with two basal body markers, centrin-4 and YL1/2, could not detect any differences between the parental and FAZ7A null mutant lines and cell lines (S5A and S5B Fig), suggesting that the basal body remains unaltered in the FAZ7A null mutant. We also verified whether FAZ7A and FAZ7B were functionally related by testing the localization of the HA-tagged FAZ7A in FAZ7B null mutants. Immunofluorescence labelling of FAZ7A in the FAZ7B KO showed that in the absence of FAZ7B, FAZ7A’s localization was unmodified and that it still localized at the basal body (S5C Fig).

As only FAZ7B, and not FAZ7A, showed a cellular localization consistent with that of other *Leishmania* FAZ proteins [4], we then focused our study on FAZ7B. We first confirmed that the growth defect in the FAZ7B null mutant was specific to this protein. For this, we exploited the CRISPR-CAS9 system to re-introduce a copy of FAZ7B tagged at the C-terminus with a Red Fluorescent Protein (RFP) in its endogenous locus. In this complemented cell line, the FAZ7B localization was checked in promastigotes (Fig 2C), and growth rates in both promastigotes (Fig 2A) and amastigotes (Fig 2B) were restored. We next tested whether FAZ7B is a functional kinesin by using the same complementation approach but introducing a mutated ATP-binding site in the kinesin motor domain. A mutated version of FAZ7B (G199A_K200A) in the highly conserved P-loop (phosphate-binding loop) motif in the nucleotide-binding domain, and C-terminally tagged with a RFP, was inserted *in situ* in the FAZ7B null mutant. A construct without the motor domain, encoding only the C-terminal part of FAZ7B with a RFP tag, was used as a negative control. Fluorescence imaging showed that both the mutated and the truncated version of FAZ7B localized at the distal part of the FP, similar to the localization observed in the wild-type and add-back cell lines (Fig 2C). Growth curves of both complementation mutants exhibited a growth defect similar to the FAZ7B null mutant, demonstrating that the kinesin motor domain is required to restore normal growth in both promastigote (Fig 2D) and amastigote stages (Fig 2E). The whole of these data (a) suggests that FAZ7B is a functional kinesin, and (b) indicates that it is required for parasite growth and (c) that the C-terminal domain is involved in targeting the protein to the FAZ region.

### Deletion of FAZ7B leads to abnormal cell division in promastigotes and amastigotes

**Fig 3:**
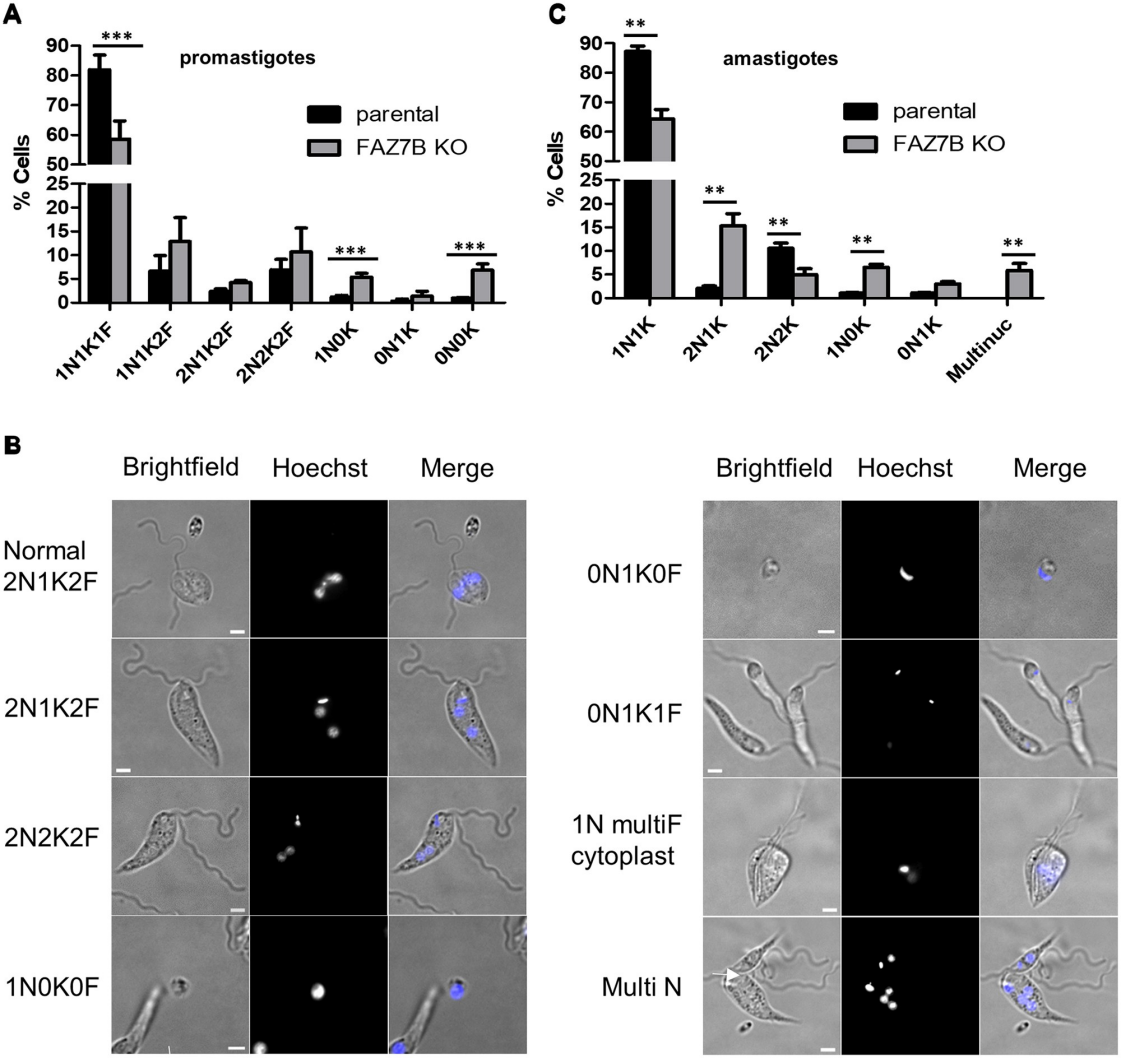
Deletion of FAZ7B impairs cell division in promastigotes and amastigotes. (A and C) Quantification of nuclei (N) and kinetoplasts (K) cell phenotypes in DAPI-stained parental and FAZ7B KO cell lines: (A) Promastigotes, (C) Axenic amastigotes. Error bars represent SDs calculated from three independent experiments (n = 300). ** p < 0.01, * p < 0.05 (Student’s t-test). (B) Examples of dividing cells in the promastigote FAZ7B KO cell line, Hoechst-stained after 14hs of time-lapse imaging, showing the indicated categories of N/K patterns; multiF: multiflagellated cell; MultiN: a cell with several nuclei and kinetoplasts; cytoplasts can be seen, with (1N0K0F and 0N1K0F) or without (left panel, top) DNA, as well as in a dividing process (1N multiF cytoplast). See also S5, S6 and S7 Movies. Scale bar: 5μm.

In *Leishmania*, cell cycle stages can be determined by analysing the number of kinetoplasts, nuclei, and flagella present in a cell, because these single-copy organelles duplicate at specific points during the cell cycle. During interphase, parasites have one nucleus, one kinetoplast and one flagellum (1N1K1F). Cell division involves first the duplication and elongation of the flagellum from a mature pro-basal body within a single FP, followed by the duplication of the latter (1N1K2F). Subsequently, nuclear mitosis and kinetoplast division occur, near synchronously in *L*. *mexicana* promastigotes, generating 2N2K2F cells [16,25]. FAZ7B null mutant cells were stained with Hoechst and N/K patterns were determined in the promastigote and axenic amastigote stages. Promastigotes of FAZ7B null mutants showed a significantly reduced number of interphasic (1N1K1F) cells (Fig 3A) and a non-significant increase in dividing cells, as well as a notable increase in ‘pseudo-cells’ termed cytoplasts (1N0K, 0N1K and 0N0K morphotypes) (Fig 3B), which were later clearly identified using live imaging (see below); this suggested strongly abnormal, though still ongoing, cell division, consistent with the observation of a decrease rather than an arrest of growth. To better analyse these cell division events, time lapse microscopy was performed during 14 hs, a time window allowing most of the control cells to undergo one or two divisions. In the parental cell line, only two events among the 106 observed divisions were abnormal, both showing one of the sister cells degenerating. In the FAZ7B mutant cell line, the rate of abnormal divisions was 33 over 67. About half of them (43%) showed abnormal cytokinesis releasing two apparently normal cells, which could eventually divide normally, and a small cytoplast generally devoid of flagellum (S6A Fig and S5 Movie). After the abnormal division, a normal round of division could restart. In 9% of the cases, the two sister cells showed incomplete cytokinesis and remained attached; some could detach later and divide again (S6 Movie). In 39% of the cases, incomplete cytokinesis was accompanied by the release of multiple cytoplasts (S6A Fig). In 9% of the cases, multiple rounds of abortive cell divisions occurred, leading to ’monster’ cells with a deformed cell body and multiple flagella, as well as cytoplasts adjacent or attached to the main cell body, and ultimately leading to cell fragmentation (S7 Movie). These ’monster’ cells were observed on DAPI-stained slides but not in sufficient numbers to make a category, probably because they are fragile and were lost during manipulation. Hoechst staining at the end of the time-lapse experiment (Fig 3B) also showed that about 50% of the cytoplasts contained DNA, but due to a high heterogeneity of the Hoechst staining, it was difficult to always differentiate the kinetoplast from nuclei or nuclei fragments. These observations were consistent with the emergence of the unusual 1N0K and 0N0K categories found in the N/K patterns (Fig 3A).

In axenic amastigotes, analysis of the cell population after Hoechst staining also showed an aberrant N/K profile but different from the one observed in promastigotes, except for the reduction in 1N1K cells. A significant increase in 2N1K cells along with reduced numbers of 2N2K cells (Fig 3C) suggested a delay in the segregation of the kinetoplast, *i*.*e*. that kDNA has replicated but the network has not been distributed into two separate daughter organelles. Significant cell division defects were also indicated by the presence of aberrant cell morphotypes, including again cytoplasts deprived of kinetoplast (1N0K) and so-called ’zoids’ (0N1K). Also, the presence of multinucleated, multi-kinetoplast and multi-flagellated cells (see below) suggested cytokinesis impairment. Taken together, these data showed that the deletion of FAZ7B caused cytokinesis defects both in promastigotes and amastigotes; but the phenotype was clearly more drastic in amastigotes.

### Deletion of FAZ7B results in altered cell morphogenesis

**Fig 4:**
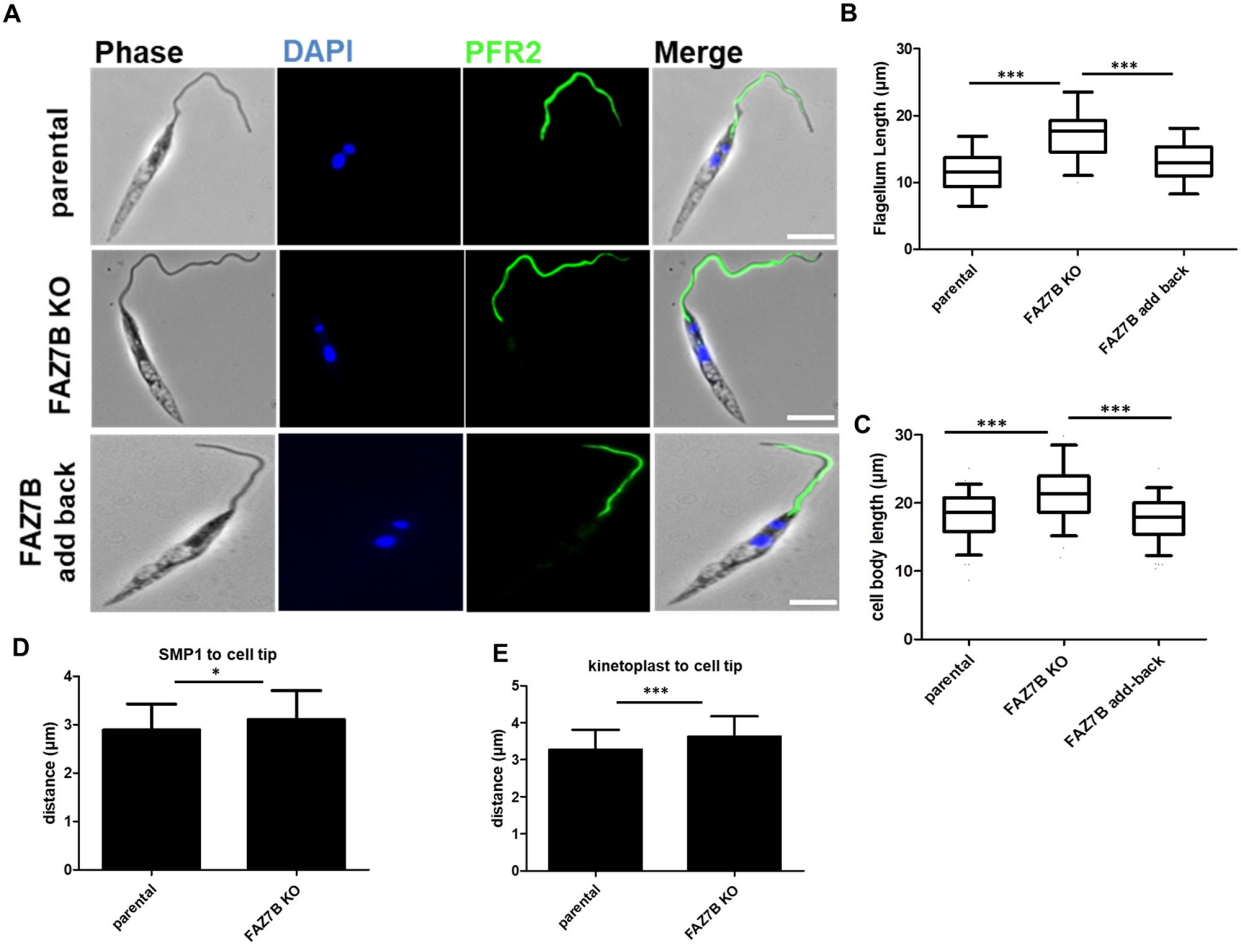
Loss of FAZ7B alters promastigote cell morphology. (A) Immunofluorescence assay. Parental, FAZ7B KO and add-back representative cells labelled using anti-PFR2 antibody (green). Scale bar: 5μm. (B) Flagellum and (C) cell body length measurements of parental, FAZ7B KO and add-back cell lines. Cells were fixed with PFA at a density of 1x10^7^ cells/mL. Boxes and error bars indicate the median, upper and lower quartiles and 95^th^ percentiles. *** p < 0.001 (Student’s *t*-test). (D-E) Measurement of the distance between the SMP1 signal (D), kinetoplast (E) and the anterior end of the cell body in parental and FAZ7B KO cell lines. One hundred cells were counted per sample. Boxes and error bars indicate the median, upper and lower quartiles and 95^th^ percentiles. *** p < 0.001; * p < 0.05 (Student’s *t*-test).

Since the FAZ has been shown to be involved in cell morphogenesis [15,16], we further analysed the cell morphology in promastigote cells. Microscopic observation of the FAZ7B null mutant revealed a greater mean flagellum length than that of the parental and add-back cell lines: 17.2±2.8 μm *vs*. 11.5±2.4 μm and 13.1±2.3 μm for the FAZ7B null mutant, parental and add-back cell lines, respectively (Fig 4B). All flagella displayed positive labelling of paraflagellar rod protein 2 (PFR2) (Fig 4A). Flagellum length was similarly increased in the double KO FAZ7A+B, while it remained unchanged in the FAZ7A null mutant (S4C and S4D Fig). FAZ7B null mutant cells also displayed a significantly increased cell body length as compared to the parental cell line (21.4±3.1 μm *vs*. 18.2±2.6 μm and 17.6±2.5 μm for FAZ7B null mutant, parental and add-back cells, respectively (Fig 4C). Previous works have shown that deletion of *Leishmania* FAZ components altered the size and shape of the FP [15,16]. To determine whether FAZ7B deletion alters FP size, we estimated FP length by measuring the distance from the proximal end of the SMP1 signal to the cell tip (the flagellum exit point from the cell body), and the distance from the kinetoplast to the cell tip. Both distances were significantly increased in the FAZ7B null mutant as compared to the parental cell line (Fig 4D and 4E). Altogether, these data show that loss of FAZ7B results in perturbed cell morphology affecting the cell body, flagellum and FP lengths.

### FP overlay and flagellum structure are preserved, but FP biogenesis is perturbed, in FAZ7B null mutant cells

**Fig 5:**
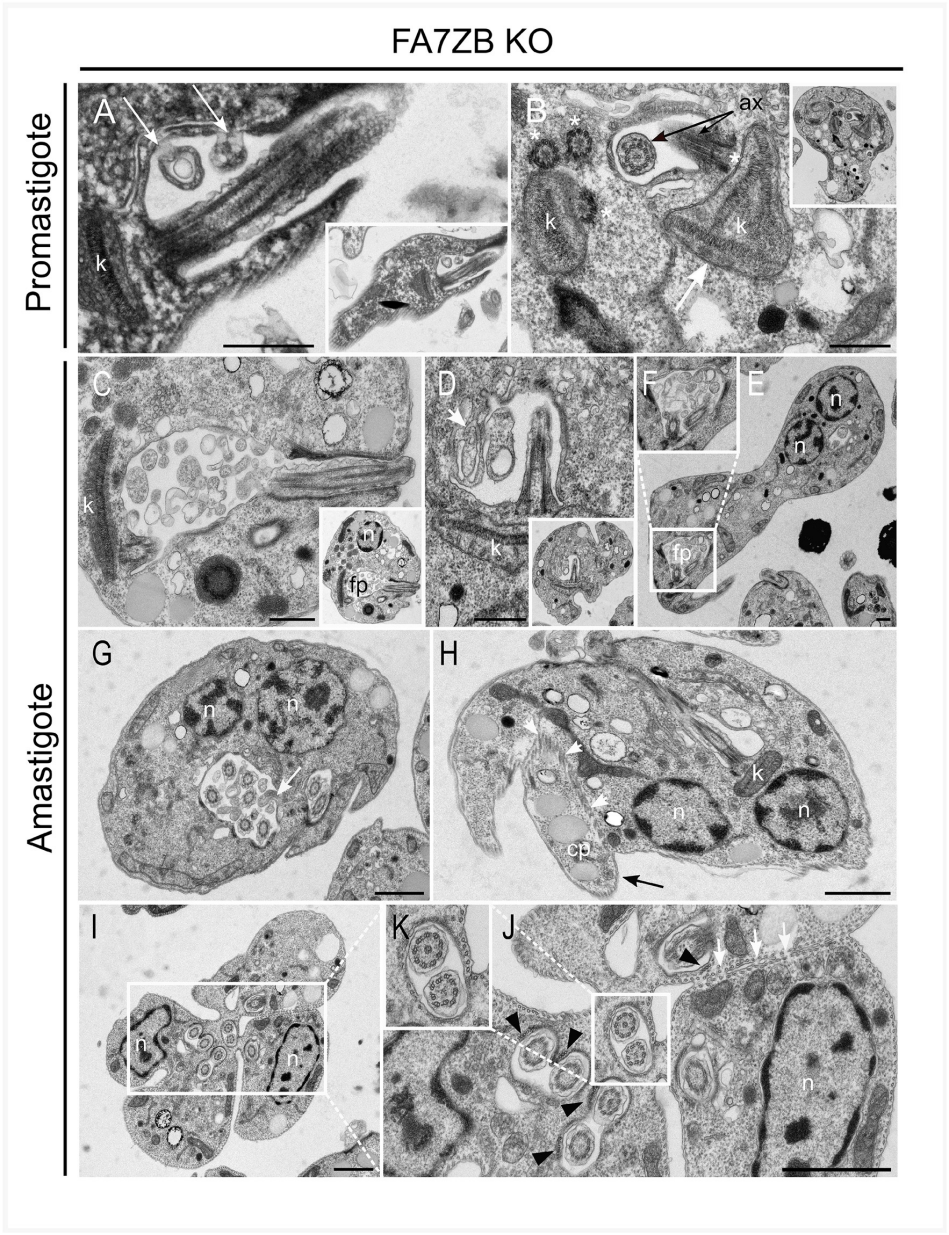
Flagellar pocket structure and cell division are perturbed in the FAZ7B null mutant. Transmission electron micrographs of promastigotes and axenic amastigotes of the FAZ7B KO cell line. Promastigotes (A-B): (A) Abnormal cytoplasmic inclusions are present in the lumen of the FP (white arrows). (B) Rare cells showed abnormal kinetoplasts and/or abnormal basal bodies: here, a mutant cell showing four kDNA networks (of which three are contigous) (white arrow) and four basal bodies asymmetrically distributed (white asterisks). The flagellum structure appears normal (ax, black arrows). Amastigotes (C-K): Abnormal cytoplasmic inclusions were frequently observed in the lumen of the FP (C-G). Depending on the cutting plane, this material could be seen as protrusions of the FP wall inside the lumen of the pocket (D, G; white arrows). (G) An amastigote cell showing two nuclei and six transversal sections of flagella in a single FP and two flagella in another one. (H) Longitudinal section of a dividing amastigote, with two nuclei and only one kinetoplast and one FP visible on this cutting plane. On the left side, a portion of cytoplasm apparently devoid of nucleus but containing lipid bodies and organites shows incomplete separation from the ‘donor’ cell, with discontinuous plasma membrane and cortical microtubules (white arrows), forming a cytoplast (cp, black arrow). (I-K) A transversal section of a dividing FAZ7B-KO amastigote showing multiple FPs each containing one or two flagella. At least three partially separated cell bodies can be distinguished. The cortical microtubule network is in place but the plasma membrane shows incomplete separation (J, white arrows). The microtubule quartet is clearly seen close to most flagella (J, black arrowheads). The flagellum structure in transversal sections appears normal. **n**: nucleus; **k**: kinetoplast; **ax**: Axoneme; **cp**: cytoplast. Scale bars: 0.5 μm.

We next examined the ultrastructural effects of the depletion of FAZ7B in promastigotes and axenic amastigotes. In transmission electron microscopy (TEM) sections of promastigote stages of the FAZ7B null mutant, the FP architecture was overall conserved, with the bulbous and neck parts clearly visible in longitudinal sections (S7A Fig). In the bulbous region of the FP, the flagellum was asymmetrically positioned within the pocket lumen as previously described [4]. At the FP neck, the well-defined electron-dense attachment areas and junctional-like complexes were clearly visible (S7A Fig). Also, the flagellum structure remained unaltered in the FAZ7B null mutant cells: the transition zone (Fig 5A), the 9+2 axonemal structure (nine outer microtubule doublets and a central microtubule pair) (Fig 5B), the extra-axonemal PFR and the MTQ were present in many flagellar sections examined. Consistent with the cytokinesis phenotype described above, a few ’monster’ cells could be seen in TEM sections, of which one showed anomalies of kinetoplast segregation with up to three kDNA networks in a single mitochondrion portion (Fig 5B).

In the FAZ7B null mutant amastigote stage, the overall FP architecture and the flagellum attachment to the cell body along the neck region of FP were also conserved (Fig 5C and S7A Fig). Consistent with the altered N/K phenotype observed for this mutant, transversal TEM sections also showed multi-kinetoplast and multinucleated (S7B Fig) cells, as well as cells comprising multiple flagella, either with their own FP or within a single large FP (Fig 5G, 5I and 5J and S7B Fig); this suggested that successive rounds of flagellum biogenesis could occur without FP division. Some cells displayed anarchic cell division with absent or incomplete sister cell separation (Fig 5H–5J and S7B Fig) and the production of cytoplasts (Fig 5H). In these abnormal amastigote cells, the flagellum structure appeared essentially conserved (Fig 5K and S7B Fig), with axonemes showing the 9+0 (9v) architecture previously described in *Leishmania* amastigotes [1]. The MTQ was also frequently observed, even in multi-flagellated cells where all flagella seemed to have their own MTQ (Fig 5J and S7B Fig).

Abnormal cytoplasmic inclusions were observed in the FP lumen of both promastigote and amastigote cells (Fig 5A and 5C–5G), suggesting a perturbation of the FP structure and function. Protrusions of the FP membrane into the lumen of the pocket were also frequently observed (Fig 5A and 5D). The frequency of these anomalies as well as their number and volume were much greater in amastigotes than in promastigotes. In our experimental set-up, it was not possible to determine if the inclusions were always in continuity with the parasite cytoplasm or if they could detach and bud into the lumen of the FP.

To go further in the analysis of the FP, we used the lipid-intercalating dye FM4-64FX to monitor plasma membrane uptake during endocytosis in interphasic cells (1N1K) by fluorescence microscopy. FM4-64FX labelling of the FP in FAZ7B null mutant cell line was reduced compared to the parental cell line (45% *versus* 85% positive labelling, respectively) (S8A and S8B Fig), indicating a delay in the incorporation of this lipophilic membrane marker (likely due to a defect in the membrane of the FP). We then labelled the bulbous part of the FP by tagging the LmxM.23.0630-Sec10 protein [15]. Considering that in *Leishmania*, the segregation of the kinetoplast likely occurs after the division of the FP [26], we focused on the late stage of cell division, namely 2N2K2F cells. In the parental cell line, two clearly distinct signals, corresponding to two FPs, were visible in 95% of the 2N2K2F dividing cells (S8C Fig). In the null mutant cell line, 40% of the 2N2K2F dividing cells displayed two FPs but with one significantly smaller than the other one; and 32% of these cells only showed a single non-segregated signal (S8C and S8D Fig). Importantly, the FP defect was always located on the short flagellum side, hence in the new daughter cell, in all cells where this could be verified. These data strongly suggest an altered/delayed division of the FP in the cells lacking FAZ7B.

### Loss of FAZ7B alters the localisation of FAZ and flagellar pocket collar proteins

The localization of FAZ7B also prompted us to test whether the loss of this protein modifies other FAZ components and/or perturbs the localization of the MTQ and FPC proteins. Components of the FAZ, MTQ and FPC were endogenously tagged with mNG in the parental and the FAZ7B null mutant cell lines. The localization of FAZ1 and FAZ10 appeared overall unchanged in the FAZ7B null mutant promastigotes (S9 Fig). By contrast, the localisation of the FAZ5 and ClpGM6 signals were not as expected. In the parental cell line, they appeared as a short line along the intracellular part of the flagellum (S9 Fig). In the FAZ7B null mutant cells, the signals were increased in length and overlapped the anterior cell tip and the proximal part of the exiting flagellum (S9 Fig). This phenotype was clearly more pronounced for ClpGM6 than for FAZ5.

We next examined the localization of the MTQ marker protein SPEF1 [9] and the FPC components BILBO1 [27] and FPC4 [28] in the FAZ7B-3xHA cell line and in the FAZ7B null mutant. In *L*. *mexicana* promastigotes, SPEF1 was reported to localize at the MTQ as well as at the multivesicular tubule lysosome and its associated microtubule [29,30]. Here, SPEF1-mNG displayed a similar localization as reported; and the FAZ7B-3xHA signal did not colocalize with, but was juxtaposed distally to, SPEF1-mNG (S10 Fig). In the FAZ7B null mutant, the localization of SPEF1-mNG was essentially unmodified (S10 Fig). As mentioned above, TEM images also showed unaltered MTQs in null mutants.

**Fig 6:**
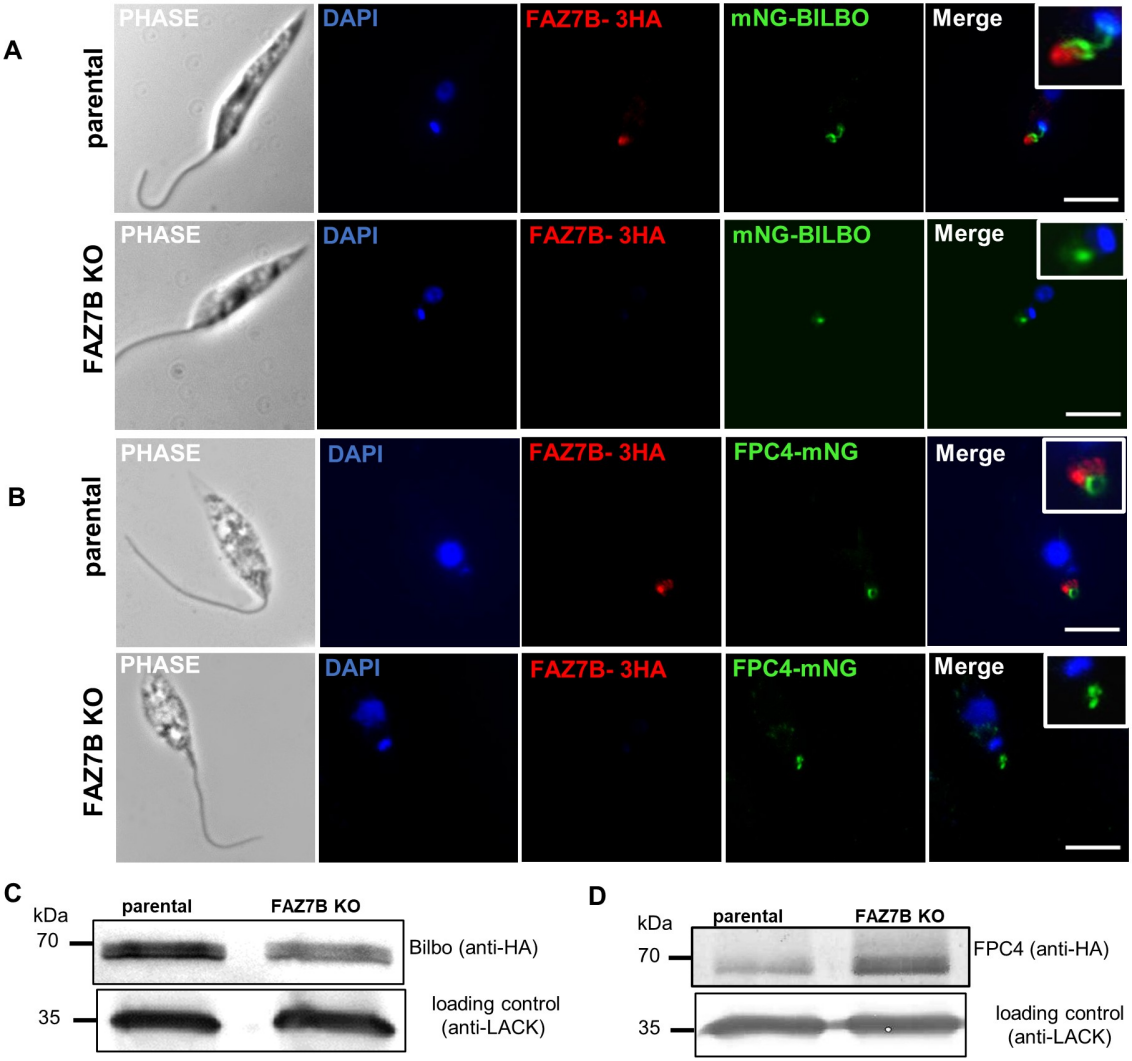
The molecular organization of the flagellar pocket collar is altered in the FAZ7B null mutant. Immunofluorescence labelling of promastigotes from parental and FAZ7B KO cell lines expressing (A) FAZ7B-3xHA (red) and mNG-BILBO1(green), and (B) FAZ7B-3xHA (red) and FPC4-mNG (green) Scale bar: 5μm. See also S11 Fig. (C-D) Western blot of promastigotes from the parental and FAZ7B KO cell lines expressing 3xHA-Bilbo (C) and FPC4-3xHA (D), using anti-HA. 5x10^6^ cells were loaded on a 12% SDS-PAGE, transferred and immuno-probed with anti-HA and anti-LACK (loading control) antibodies.

BILBO1 in *T*. *brucei* is a scaffold protein of the FPC and is essential for FP and FPC biogenesis and cell survival [5,6]. In the *L*. *mexicana* FAZ7B-3xHA cell line, the mNG-BILBO1 signal was often contiguous with FAZ7B and visible as a horse-shoe, continued with a helix shape joining the kinetoplast which in *T*. *brucei* is the MTQ [28] (Fig 6A and S11A Fig). However, in the FAZ7B null mutant, the ring and the helix structure were lost; and only a single short line or dot without any link to the kinetoplast was observed (Fig 6A and S11A Fig). This suggests that the deletion of FAZ7B displaces BILBO off the FPC/MTQ structure and alters the organization of the FPC in *L*. *mexicana*.

TbFPC4 is a TbBILBO1 partner and a microtubule binding protein that is involved in FPC segregation [28]. In the LmxFAZ7B-3xHA cell line, the FPC4-mNG signal appeared as a ring at the anterior end of the cell, distal and often contiguous to FAZ7B-3xHA (Fig 6B); but in the FAZ7B null mutant, the ring structure was altered and the FPC4-mNG signal was disorganized (Fig 6B and S11B Fig). To analyse whether deletion of FAZ7B also modified the expression of these FPC proteins, we first attempted endogenous 3xHA tagging of both alleles of BILBO1 and FPC4, independently, in the parental and FAZ7B null mutant, using two drug resistance markers. While tagging of BILBO1 with one selective marker was achieved in the transfected parasites, we were unable to generate parasites with both alleles tagged. FPC4 tagging using two selective markers was successful and the absence of wild-type allele in the cell population was verified by PCR (S3E Fig). Western blot analysis with an anti-HA antibody showed different levels of expression for BILBO1 and FPC4 in the parental and FAZ7B KO cell lines. Indeed, densitometric analysis of the bands showed a 40% reduction and a twofold increase of the protein levels of BILBO1 and FPC4, respectively (Fig 6C and 6D), in the FAZ7B null mutant compared with the parental cell line. Taken together, these data suggested that deletion of FAZ7B alters the protein levels of BILBO1 and FPC4 and the molecular organization of the FPC.

### Ablation of FAZ7B, but not FAZ7A, affects promastigote motility and development in the sand fly vector

**Fig 7:**
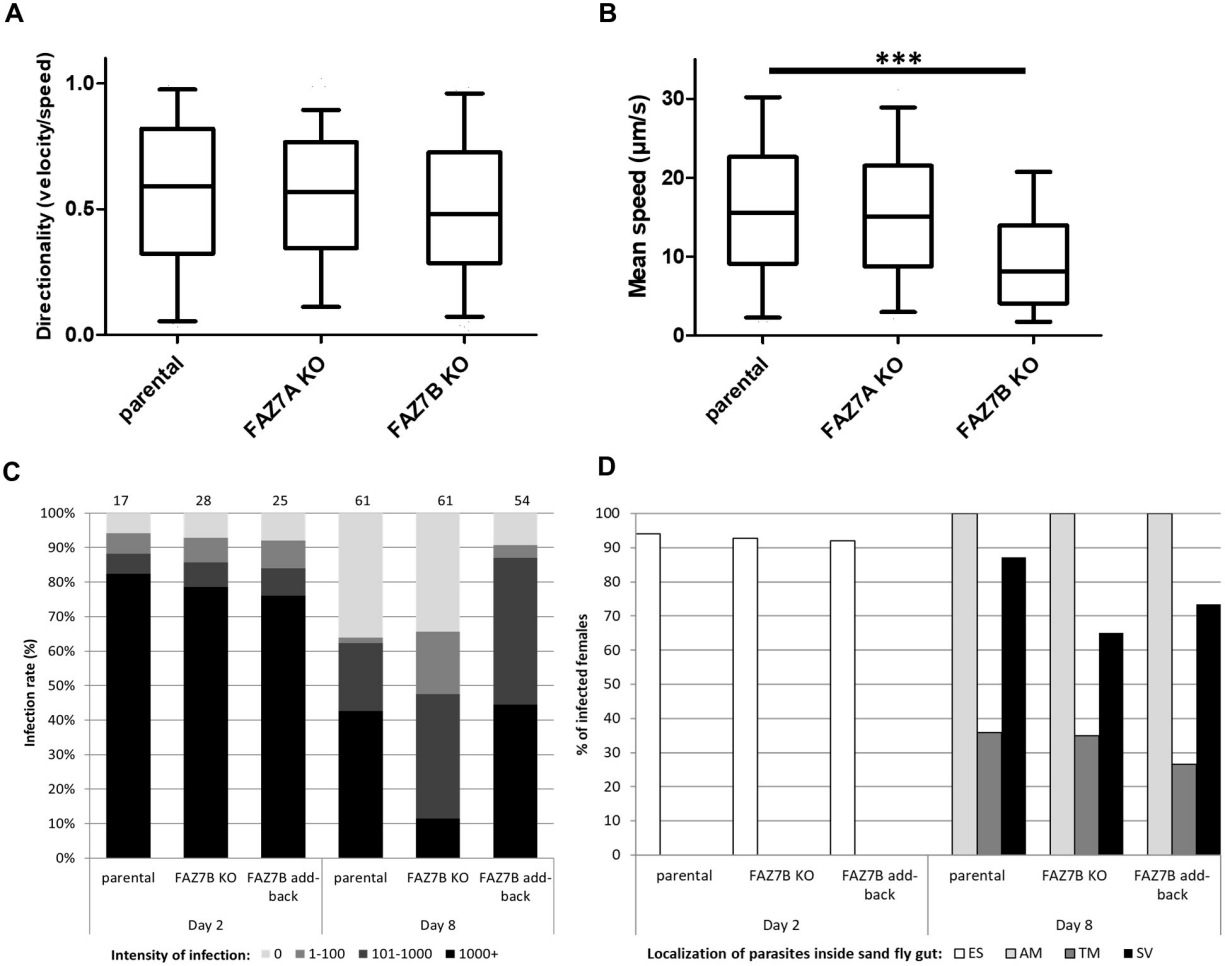
Deletion of FAZ7B impairs motility, proliferation and development of *Leishmania* in the sandfly. (A) Directionality (velocity/speed) and (B) swimming speed measurements of parental, FAZ7A KO and FAZ7B KO promastigotes were calculated from swimming tracks obtained from video microscopy using the plugin Trackmate. Boxes and error bars indicate the mean, upper and lower quartiles and 95th percentiles. *** p < 0.001 (Student’s *t*-test). (C) Infection rate and intensity of infection in *Lutzomyia longipalpis* females infected with parental, FAZ7B KO and FAZ7B add-back cells. After 2 days and 8 days post-blood meal (PBM), sandflies were dissected (numbers indicated above the column) and the parasite load in each fly was measured as weak (1–100 parasites), moderate (101–1000 parasites), or heavy (1000+ parasites). The differences between the sandfly infections caused by parental and KO cell lines on day 8 PBM were highly significant (Chi-squared test, X^2^ value: 22.237, p value: 7.02.E-7). (D) Localization of *Leishmania* parasites within infected sandflies after 2 days and 8 days PBM. ES: endoperitrophic space, AM: abdominal midgut, TM: thoracic midgut, SV: stomodeal valve. Differences were observed among infections caused by the three cell lines but were not found statistically significant.

Given the altered flagellum length in the FAZ7B null mutant, we next investigated whether motility was affected in this mutant. Time-lapse were performed and analysed using the Fiji plugin TrackMate [31]. The directionality (velocity/speed), a measure of displacement from a starting point to another one over time, was similar between the parental and FAZ7B null mutant cell lines (Fig 7A). However, the speed was significantly reduced in the FAZ7B null mutant cell line (9.17 ± 4.9 μm s^-1^) compared to the parental cell line (16.04 ± 7.6 μm s^-1^) (Fig 7B and S8 and S9 Movies). It is noteworthy that this modification was not observed in the FAZ7A null mutant, for which no significant differences were detected in the directionality and speed compared to the parental cell line (Fig 7A and 7B).

We then investigated whether these changes could modify the capacity of FAZ7B null mutant to proliferate and develop in the insect vector. Indeed, flagellum attachment to the sand fly midgut epithelium is crucial for the life cycle of the parasite to proceed [32]. We infected female *Lutzomyia longipalpis* sandflies with the parental, FAZ7B null mutant and add-back cell lines. The sandflies were then dissected before defecation on day two post-blood meal (PBM) and after defecation on day eight PBM. Early-stage development resulted in similar infection rates (92–94%) and similar infection intensities for all cell lines tested. Procyclic promastigotes were located inside the endoperitrophic space, within the blood meal surrounded by the peritrophic matrix. However, the late-stage infections significantly differed both quantitatively and qualitatively. The intensity of infection was significantly higher in females infected with parental and complemented cell lines: for these, a heavy/medium infection dominated; conversely, a lower intensity of infection was observed in flies infected with FAZ7B null mutants (Fig 7C). The localization of parasites in the sandfly midgut at the late stage of infection also differed, *i*.*e*. more frequent colonization of the stomodeal valve was observed for the parental (87.2%) and add-back cell lines (73.5%) compared to the FAZ7B null mutant (65%); yet, these differences were not found statistically significant (Fig 7D). These data showed that deletion of FAZ7B impaired proliferation of promastigotes in the insect vector, hence possibly a successful transmission.

### Cells lacking FAZ7B display reduced pathogenicity in mice

**Fig 8:**
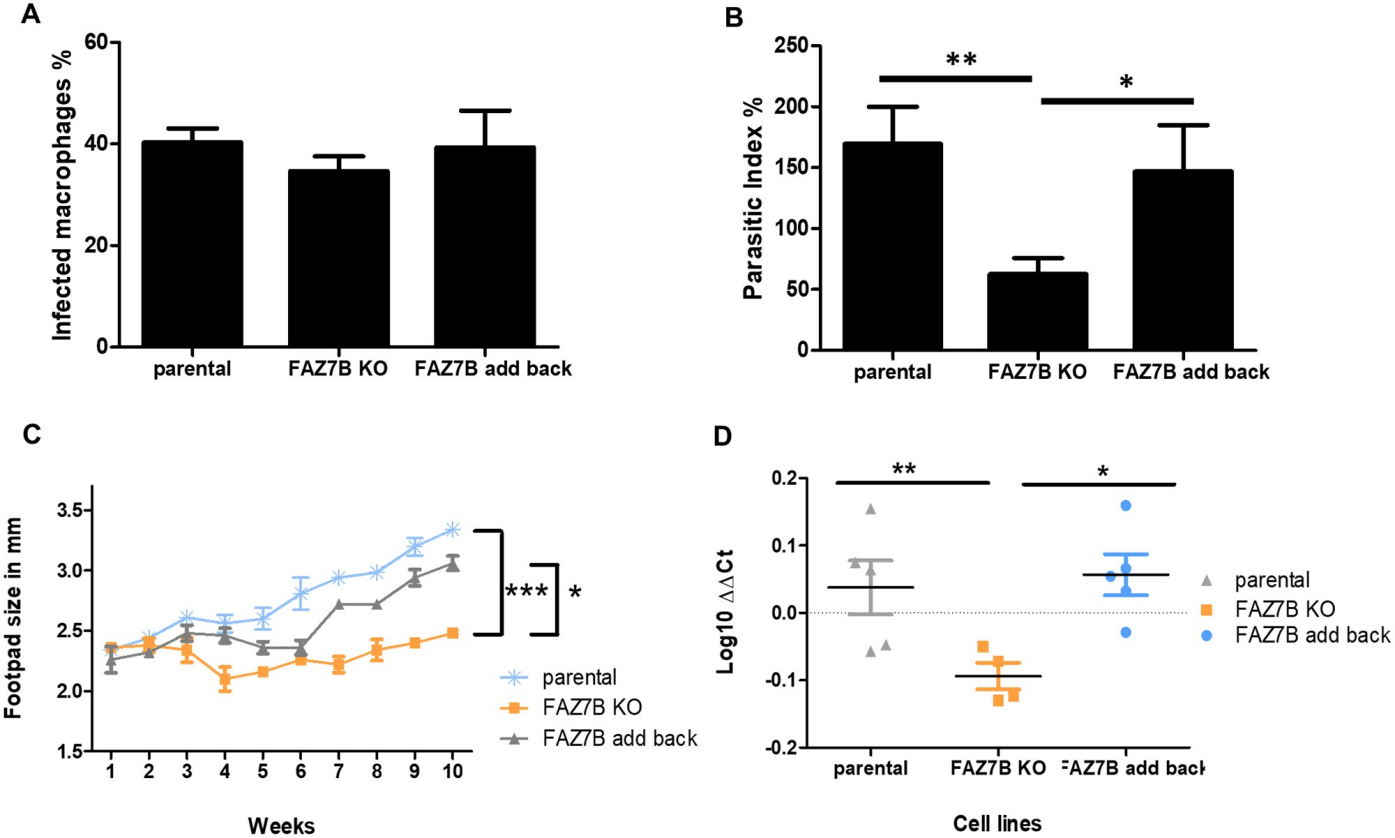
Loss of FAZ7B reduces pathogenicity in the mouse. (A-B) Axenic amastigotes of parental, FAZ7B KO and FAZ7B add-back cells were used to infect differentiated THP-1 monocytes. Percentage of infected macrophages (A) and parasitic index (B) were determined at 72h post infection by Giemsa staining as described in Experimental procedures. The parasitic index (PI) was defined as the percentage of infected macrophages x number of intracellular parasites/macrophage. ** p < 0.01, * p < 0.05. (C) Measurement of mean footpad lesion size during a ten-week infection time course with parental, FAZ7B KO and FAZ7B add-back stationary-phase promastigotes. Error bars represent SDs. The *p* value was calculated using two-tailed unpaired Student’s *t*-test comparing each cell line at week 10. *** p < 0.001, * p < 0.05. (D) Relative amount of *Leishmania* DNA compared to mouse DNA by qPCR at 10 weeks post-infection by parental, FAZ7B KO and FAZ7B add-back cells. ** p < 0.01, * p < 0.05 (Student’s *t*-test).

We next analysed the ability of the parental, FAZ7B null mutant and add-back cell lines to infect and replicate within THP-1-differentiated monocytes. All cell lines displayed similar infection rates at 72 hours post-infection (Fig 8A), but the ability of FAZ7B null mutant parasites to proliferate inside macrophages was significantly impaired, as shown by the strong reduction of the parasitic index in the FAZ7B null mutant (Fig 8B).

We then tested whether the FAZ7B null mutant had an altered pathogenicity/virulence in a mouse model for cutaneous leishmaniasis. Footpad measurements over a ten-week period indicated that FAZ7B null mutants did not form significant lesions as compared to the parental and add-back cell line (Fig 8C). Quantitative PCR of parasite burden using primers for *Leishmania* kinetoplast DNA minicircle and mouse 5.8S RNA showed significant differences between the relative amount of *Leishmania* DNA for FAZ7B null mutants and those for the parental and add-back parasites (Fig 8D). No significant differences were detected between the data for the parental and complemented cell lines. Altogether, these data showed that *L*. *mexicana* lacking FAZ7B was able to infect macrophages *in vitro* but with a reduced proliferation rate, and exhibited a reduced pathogenicity and virulence *in vivo*, causing smaller lesions and a lower parasite burden than the parental cell line.

## Discussion

The trypanosomatid genomes encode 55 kinesin-like proteins, with FAZ7 being the only known FAZ protein containing a kinesin motor domain. In *T*. *brucei*, FAZ7 is located at the distal end of the FAZ region and its function is yet unknown [20]. The gene is duplicated in *Leishmania*, and both paralogs share low sequence similarity and differ in their localization: FAZ7A localizes at the basal body, while FAZ7B localizes at the neck of the FP where the FAZ is present in *Leishmania* [4]. In addition, we show here that both paralogs play distinctive and non-redundant roles: in contrast to FAZ7A, deletion of FAZ7B affected growth rate, cell division, cell morphogenesis and motility. The low protein identity (28% without the motor domain sequence) between FAZ7A and FAZ7B further supports different functions for these paralogs in *Leishmania*. Other examples of kinesin paralogs having distinct functions but only one required for cell survival have been previously reported in trypanosomatids [33,34]. Here, given the extensive repertoire of kinesins in the trypanosomatids, it is not surprising that the duplication of FAZ7 in *Leishmania* resulted in two kinesins playing independent roles: FAZ7A is a basal body protein dispensable for cell growth, whilst FAZ7B is at the cell body side of the FAZ and is important for parasite proliferation. Our data thus also highlight the diversity and high specificity of functions among the large repertoire of the trypanosomatid-specific kinesins.

Complementation of the FAZ7B null mutant with a mutated version of the motor domain, or with the C-terminal sequence lacking the kinesin motor domain, was unable to restore growth rate. Yet, these truncated proteins were able to localize at the distal part of the FP. These data suggest (i) that FAZ7B is a functional kinesin; and (ii) that, like most kinesins, the targeting to the FAZ region is independent of the motor domain sequence and related to the more divergent (C-terminal) part of the protein [35].

Localization studies on FAZ7B strongly suggest that FAZ7B is a component of the cell body cytoskeletal region of the FAZ, as opposed to it being in the flagellar cytoskeleton domain. This is indicated by fluorescence microscopy showing proximity of FAZ7B to FAZ proteins of the cell body domain (Fig 1B, S2B and S2C Fig), as well with proteins of the flagellar collar (Fig 6A and 6B). The apparent proximity of FAZ7B to the MTQ (S10 Fig) also suggests localization of the kinesin on the cell body side of the FAZ. Yet, FAZ7B being a kinesin, it is unclear to which microtubules this kinesin may be linked in this region. Given that FAZ7B does not colocalise with SPEF1, a marker of the MTQ [29,30], and that SPEF1 was not affected in the mutant lacking FAZ7B, it is unlikely that FAZ7B is part of this structure. Hence, FAZ7B might be associated to a specific part of the cortical microtubules, or alternatively to the so-called cytoplasmic microtubules or the pocket microtubules [4] whose functions are unknown.

Deletion of FAZ7B resulted in two major phenotypes: altered morphogenesis (including the flagellum, FP and cell body); and disruption of cytokinesis. Among the many intertwined factors involved in cell morphogenesis [36,37], the FAZ plays a major role. Indeed, in the related parasite *T*. *brucei*, besides connecting the flagellum to the cell body, the FAZ is involved in cell size and shape control [7,20]. Hence, the FAZ has been proposed as a ’cellular ruler’ for trypanosome morphology [7]. Recent works revealed that, in *Leishmania*, FAZ components are also involved in FP structure and biogenesis and maintenance of cell morphology [15], as well as cell tip morphogenesis [16]. Contrary to the deletion of *Leishmania* FAZ5, which resulted in shortened cell body, FP and flagellum [15], the KO of FAZ7B here resulted in a significant increase in length of these same parameters. Moreover, the formation and function of the FP were perturbed, as could be seen (i) in TEM from the presence of multiple flagella in single FPs in axenic amastigotes, and from cytoplasmic material in the FP in both lifecycle stages, and (ii) in fluorescence microscopy from a disruption of the FP division in promastigotes. These data are in agreement with a key role of FAZ components for FP morphology and biogenesis in *Leishmania*.

Moreover, the molecular organization of the FPC is perturbed in the FAZ7B null mutant, as suggested by the altered subcellular localization of the FPC components BILBO1 and FPC4. TbBILBO1 is a protein of the FPC; its depletion in *T*. *brucei* induces flagellum detachment and perturbs the biogenesis of the FP, FPC and the FAZ [5,6,27], showing the tight inter-dependency between these different structures/processes. Although BILBO1 remains uncharacterized in *Leishmania*, it is likely to play similar essential functions in this parasite. Here, despite the slightly reduced protein levels and modified localization of BILBO1 in the FAZ7B null mutant, the FPC could be seen is some cells in TEM; this suggests a disruption of the link between the collar and the MTQ rather than a complete disruption of the collar itself. Thus, we cannot determine how the phenotype caused by FAZ7B deletion in *Leishmania* is related to BILBO1, whether through its displacement or through a partial loss of the protein or through a perturbation of its function; nevertheless, this may participate in the altered FP biogenesis and subsequent cell division defects.

The deletion of *Leishmania* FAZ components alters the localization of various FAZ proteins [15,16]. The ablation of FAZ5, a multimembrane FAZ protein involved in the attachment of the cell body membrane to the flagellum membrane, induces the uncoupling of both membranes, as shown by a disorganized fluorescence signal of FAZ1, FAZ2 and ClpGM6 [15]. The deletion of FAZ7B here induced a mis-localization of both FAZ5 and the flagellum side FAZ protein ClpGM6, also suggesting a disruption of the link between the cell body and flagellum domains of the FAZ. This phenotype was also reminiscent of the changes described after knocking out the LmxFAZ2 protein, where ClpGM6 and FAZ5 also re-localized along the flagellum, within an extension of the anterior cell tip [16]. However, there are major differences in the consequences of the KO of these three proteins: (i) the cell body, FP and flagellum are shortened in the FAZ5 null mutant but lengthened in the FA7B KO, and unaltered in the FAZ2 KO; (ii) deletion of FAZ5 and FAZ2 does not clearly affect cell proliferation in culture, while deletion of FAZ7B results in a growth defect in promastigotes and a growth arrest in axenic amastigotes; (iii) the flagellum attachment is strongly altered in the FAZ5 null mutant, only mildly compromised in the FAZ2 KO and apparently unaltered here in the FAZ7B KO, indicating that flagellum attachment to the FP neck membrane is independent of FAZ7B. This suggests that, if FAZ7B may be involved in the structural cohesion of the cytoplasmic versus flagellar FAZ domains, it clearly participates in a distinctive manner to the FAZ function.

*Leishmania* FAZ7B is required for proper parasite proliferation, and its ablation leads to perturbations of cell division. Multinucleated and multi-flagellated cells were observed both in promastigotes and axenic amastigotes of the FAZ7B null mutant, indicating defects in the final stage of cell division. Several factors might contribute to these cytokinesis defects. At the anterior of the cell, trypanosomatids contain a series of structures tethered in a continuous array linking membranes and cytoskeletal organelles [3,9]. Consequently, the duplication and segregation of the different single-copy organelles during cell division must be strictly coordinated both spatially and temporally. In *T*. *brucei*, the FAZ and the FP participate to this strict segregation order as they are both required for the correct positioning of these organelles before (and after) cytokinesis [12,38,39]; moreover, FP duplication is part of the sequence of coordinated events required for normal cytokinesis to proceed [38,40]. In *Leishmania*, FP biogenesis is ill-known, but the duplication of the FP is linked to the duplication and segregation of the kinetoplast [2,26]. Thus, a delay or perturbation of FP biogenesis may lead to the anomalies observed in kinetoplast segregation, and to the disruption of cell division. Finally, in *T*. *brucei*, correct FAZ assembly is important for determining the site of cytokinesis initiation [12,41]; this function is suspected [7] but yet to be explored in *Leishmania*.

The phenotype observed after deletion of FAZ7B is more pronounced in the axenic amastigote than in the promastigote life cycle stage. This is consistent with the increased expression of FAZ7B in this stage and perhaps a more important role of FAZ7B in the amastigote. Several hypotheses may be envisaged for this observation. First, the amastigote FP neck is dominated by the FAZ structures [4] which might determine the opening and the access of molecules to the FP. Differential opening of the FP between promastigote and amastigote stages may be linked with the interest for the parasite to tightly control exchanges with the exterior in the hostile mammalian host environment [4,42]. Another possibility may be that the sequence of events required for cell division is different in amastigotes, for example with a more central role of FP biogenesis in the orchestration of cell division. Alternatively, the cellular cytoskeleton might be more important for cytokinesis in the absence of a motile flagellum. Indeed, this may be compared with null mutants of the KHARON complex [43,44] who show a cytokinesis defect specifically in the intracellular amastigote stage. The KHARON complex localizes to the cortical microtubules; it is thus tempting to speculate that FAZ7B might play a role directly or indirectly in the interaction between the FAZ and cortical microtubules. In total, it remains to be determined if FAZ7B is involved in cytokinesis either directly or through the FAZ, or if the cytokinesis defects observed in the null mutant indirectly result from a perturbation of the cascade of events leading to correct cell division, in particular an altered FP biogenesis.

Promastigote flagellum length modifications and altered cell motility may be secondary, and indirectly related to the extensive perturbations of the cell cycle in FAZ7B null mutants [25]. This might also be attributed to a possible relationship between the FAZ and the definition of the flagellar beat plane, its perturbation possibly altering swimming behaviour [25]. In the sandfly, promastigotes of the FAZ7B null mutant are able to proliferate in the midgut of the sand fly (early stage of infection); but their development is impaired in late-stage infections. It is noteworthy that the loss of FAZ5, FAZ2 [15,16] and now FAZ7B in *Leishmania* affects motility and proliferation in the insect vector. This is in keeping with the fact that motility is essential for *Leishmania* to complete its life cycle in the sandfly [32,45].

Deletion of FAZ7B also reduces the pathogenicity in mice, causing reduced lesions and a lower parasite burden. Promastigotes of the FAZ7B null mutant were able to infect macrophages and differentiate into intra-cellular amastigotes *in vitro*. Hence the reduction in pathogenicity in mice is likely caused by a reduction in amastigote proliferation mediated by the drastic defect in cell division observed in this stage. These data strongly suggest that paradoxically, null mutant parasites retain their ability to invade macrophages but are not able to proliferate. Reduced pathogenicity in mice has also been demonstrated in the *Leishmania* FAZ5 and FAZ2 null mutants and this phenotype was in part associated to the changes in the FP morphology in these parasites [15,16]. Following the depletion of FAZ7B, the cytokinesis defects and reduced growth rates are added to the FP changes, which can explain the near absence of proliferation in mice.

In summary, we demonstrated here that deletion of the kinesin FAZ7B disrupts cell division, cell morphogenesis and proliferation in the sandfly and mouse. Although the function of the FAZ is ill-known in *Leishmania*, these data further underpin the essential role of FAZ components as factors required for a successful life cycle from the sandfly to the mammalian host. Albeit *Leishmania* possess a rudimentary and ancestral FAZ structure [46], it now clearly appears that the FAZ components may differentially contribute to diverse functions of the FAZ [15,16]. Thus, FAZ7B might participate in part of the FAZ functions; this might include cell morphogenesis and possibly FP biogenesis, and/or a role in the interaction between the FAZ and microtubules. Further studies of FAZ7B and other FAZ proteins and the different sets of microtubules in this region will help understanding the complex relationships between the composition, structure and function of this intriguing cytoskeletal complex.

## Materials and methods

### Ethics statement

Experiments involving mice were conducted according to the Animals (Scientific Procedures) Act of 1986, United Kingdom, and had approval from the University of York Animal Welfare and Ethical Review Body (AWERB) committee.

### Cell culture

*L*. *mexicana* promastigote forms of *L*. *mexicana* Cas9 T7 strain [21] (derived from *L*. *mexicana* WHO378 strain MNYC/BZ/62/M379) were grown at 27°C in HOMEM medium supplemented with 0.005% hemin and 10% FCS [47]. For selection and maintenance of genetically modified *L*. *mexicana Cas9 T7* lines, the relevant selection drugs were added to supplemented HOMEN medium as previously described [21]. Axenic amastigotes were generated by subculturing into Schneider’s Drosophila medium (Thermo Fisher) supplemented with 20% FCS and 25mM MES-HCl (pH 5.5) at 34°C with 5% CO2 and grown for 72 h without subculture.

### Generation of *L*. *mexicana* transgenic cell lines

Deletion of *FAZ7* genes (*FAZ7A*: LmxM.19.0680 and *FAZ7B*: LmxM.19.0690) and tagging was performed using CRISPR-Cas9 as previously described [21]. The online primer design tool www.LeishGEdit.net was used to design primers for amplification of the 5’ and 3’ sgRNA templates and for amplification of donor DNA from pT, pPLOT and pLPOT plasmids [21,29].

### DNA constructs

All primers used in this study are listed in S1 Table. FAZ7B full length and the C-terminal sequence missing the codon stop was amplified by PCR and cloned in fusion with the Red Fluorescent Protein into pNUS-RFPcD (www.pnus.cnrs.fr) [48] using *Nde*I and *Kpn*I cloning sites. Site-directed mutagenesis was directly performed on pNUS-RFPcD_*FAZ7B_WT* using the In-Fusion HD Cloning Kit (Clontech) with self-complementary primers to obtain pNUS-RFPcD_*FAZ7B_G199A_K200A*. To knock-in the constructs in FAZ7B null mutants using CRISPR-CAS9, a ~130-bp fragment of 5’UTR and 3’UTR of *FAZ7B* was cloned in the constructs pNUS-RFPcD_*FAZ7B_WT*, pNUS-RFPcD_*FAZ7B_G199A_K200A* and pNUS-RFPcD_*FAZ7B*_C-term with the in-fusion cloning kit using *Hind*III and *Pci*I cloning sites. A *Hpa*I site was added to the forward primer for 5’UTR cloning and a *MfeI* site added to the reverse primer for the cloning of the 3’UTR fragment. The resulting plasmids were digested with *Hpa*I and *Mfe*I and then ethanol precipitated before transfection. Primers for amplification of the 5’ and 3’ sgRNA templates for knock-in of the final constructs in FAZ7B null mutant cell line are listed in S1 Table. All DNA constructs were verified by DNA sequencing prior to transfection.

### Western blot analysis

For western blotting analysis, either 5x10^6^ cells were loaded per lane or equal concentrations of protein extract as quantified by Bradford assay were separated by SDS-PAGE and transferred onto PVDF membranes (Hybond-P, Amersham). Primary antibodies against HA epitope (Roche) were used at a 1:2000 and anti-LACK was used as a loading control at a 1:5000 concentration. Membranes were incubated with Alkaline Phosphatase conjugated secondary rat or rabbit antibodies (Promega) at 1:2500 and 1:7500 dilutions respectively for 1 h at room temperature, and revealed with 5-bromo-4-chloro-3-indolyl phosphate/nitro blue tetrazolium (BCIP/NBT; Promega) according to manufacturer’s instructions. Densitometric measurements for quantitation of bands were performed using ImageJ (https://imagej.nih.gov/ij/). All measurements were normalized using LACK as a loading control.

### Fluorescence microscopy

For immunofluorescence, whole cells were washed in PBS, then spread on poly-lysine–coated slides. For cytoskeleton preparations, cells were first extracted with 0.25% NP40 in PIPES buffer (100 mM PIPES [pH 6.9], 1 mM MgCl2) for 5 min, and then washed twice in PIPES buffer. Cytoskeletons were fixed overnight in cold methanol at -20°C. For whole cells, slides were fixed for 5 min in 3% paraformaldehyde and neutralised for 10 min in 100mM Glycine, then permeabilized in 0.2% Triton X100 for 10 min. After two washes in PBS, whole cells and cytoskeletons were blocked in 1% bovine serum albumin (BSA) for 30 min. Cells were probed with anti-PFR2 (2E10B7) 1:2000, anti-HA (Sigma) 1:200, anti-mCherry (Gene Tex) 1:1000, anti-YL1/2 (Millipore) 1:1500 and anti-mNeonGreen (Chromotek) 1:1000 in 1% BSA overnight at 4°C. Slides were washed in 1% BSA before incubation with anti-IgG specific secondary antibodies conjugated to Alexa Fluor 488 (1:4000) or Alexa Fluor 546 (1:2000) (Invitrogen) for 1 h. Slides were washed in PBS, and DNA was stained with DAPI and mounted with Slowfade (Molecular Probes). Images were acquired using MetaView (Universal Imaging) software, on a Zeiss Axioplan 2 microscope equipped with a Photometrics CoolSnap CCD camera (Roper Scientific). Flagellum length measurements and processing of images were completed in ImageJ (https://imagej.nih.gov/ij/).

### Live cell imaging

Live imaging was performed on a Zeiss Axiobserver Z1 on the MRI-DBS Imaging platform equipped with an incubation chamber set at 27°C and a Cool Snap HQ 2 camera. For the 14 hours time-lapse, cells were semi-immobilized in 0.5% low-melting agarose in a chamber slide (Ibidi), and imaged using bright field illumination with a 63X/1.4 Oi1 apochromat objective. Automatic image acquisition was done every 10 min on 20 positions per cell line on a single medium plane using Zen Software (Zeiss). The focus was maintained using definite Focus. After completion of the assay, the nuclei were stained using Hoechst33342 which was added in the wells, and large mosaic images were acquired. Images were analysed with ZEN.

For the motility assay, cells taken from cell cultures at a density of 5x10^6^ cells/mL were used. 5 μL of cell culture were placed on a glass slide covered with a #number 1.5H coverslip and imaged 30 sec at five frames per second using TL bright field illumination with a 10X/0.25 Ph 1 objective. Mean speed and directionality (Velocity/Speed) were measured using the open source Fiji plugin TrackMate [31].

### 3D-SIM super-resolution imaging

3D Structured Illumination Microscopy images were acquired using an OMX-V4 (GE Healthcare) equipped with a 100x/1.4NA objective, with lasers emitting at 488nm and 561nm for the green and red fluorophores respectively as well as the adequate emission filters (528/48 and 609/37). Images were reconstructed using SoftWoRx (GE Healthcare). As the labelling was well contrasted and photo-stable, no special parametrization was needed and the default wiener filter (0.001) and bias offset (65) were used. Optical Transfer Functions where experimentally measured for each individual wavelength according to manufacturers’ instructions.

Co-localization between the green and red signals was calculated using the Pearson coefficient and van Steensel randomization method. This method quantifies the Pearson cross correlation coefficient after progressively shifting one of the channels in x by 20 pixels in each direction. Co-localization was measured in the mid-z-section of each of the images.

### Transmission electron microscopy

Cells were fixed for 2 h at room temperature with 2.5% glutaraldehyde in 0.1 M cacodylate buffer (pH 7.4), and then stored at 4°C until further processing. Samples were then washed with cacodylate buffer, and fixed for 1 h in 1% osmium tetroxide in the same buffer, followed by overnight incubation in 2% uranyl acetate. Dehydration was performed in acetonitrile series, and samples were impregnated with epon 118-acetonitrile (50:50) and then 100% epon 118. Polymerization was performed at 60°C for 48 h. Ultrathin 70-nm sections were cut with a Leica UC7 Ultramicrotome, counterstained with uranyl acetate and lead citrate, and observed in a JEOL 1200 EXII transmission electron microscope at Electron Microscopy facility of the University of Montpellier. All chemicals were from Electron Microscopy Sciences, and solvents were from Sigma.

### Uptake of FM4-64FX

Microscopic analysis of FM4-64FX uptake was carried out as previously described [5] with slight modifications. Briefly, exponentially growing promastigotes (5x10^6^ cells) were incubated 10 min in the dark at 27°C in complete HOMEM medium with 10μg.mL^-1^ FM4-64 (Molecular Probes). After incubation, 1 mL of cold PBS was added to stop up-take. Cells were fixed with cold 2% paraformaldehyde solution in PBS at 4°C during 5 min. Cells were resuspended in 100 μL PBS with 10μg/mL Hoechst 33342 and imaged immediately on poly-lysine coated slide.

### Macrophage infections

Human leukemia monocyte cells (THP-1 cells) were used for *in vitro* infection as previously described [49]. Briefly, THP-1 cells in the log phase of growth were differentiated in Labtek chamber slides at a concentration of 5 x 10^4^ cells/well by incubation for 2 days in medium containing 20 ng of phorbol myristate acetate/mL. THP-1 cells treated with PMA were infected with axenic amastigotes at a parasite/macrophage ratio of 10:1 for 4 h at 37°C with 5% CO_2_. Non internalized parasites were removed. After three days of incubation, cells were fixed with methanol and stained with Giemsa. The parasitic index (PI) was defined as the percentage of infected macrophages x number of intracellular parasites/macrophage.

### Sand fly infections

A sandfly colony of *Lutzomyia longipalpis* was maintained under standard conditions, as previously described [50]. Before infections, parasites were washed three times in saline solution and resuspended in defibrinated heat-inactivated sheep blood at 10^6^ promastigotes per millilitre. Sandfly females, 3–5 days old, were fed through a chick skin membrane [50]. Fully engorged females were separated and maintained at 26°C with free access to 50% sucrose solution. Dissections were performed before defecation (early stage of infection) on day 2 post blood meal (PBM) and after defecation (late stage of infection) on day 8 PBM. Abundance and localization of parasites in the sandfly midgut were examined by light microscopy and parasite loads were graded as light (less than 100 parasites per gut), moderate/medium (101–1000 parasites per gut) and heavy (more than 1000 parasites per gut) as previously described [51]. The experiment was repeated three times. Comparison of parasite infections and localizations inside the sandfly midgut on day 8 PBM for the three cell lines was evaluated by the Pearson’s Chi-squared test.

### Infection of mice

Strain virulence was assessed by footpad swelling and quantitative PCR of footpad. For experimental infections, the parasites had previously been passaged through mice, isolated, and transformed into promastigotes before re-infection. Groups of five female BALB/c mice (6 weeks of age) were infected subcutaneously, in the left footpad, using 2×10^6^ cells from a three day stationary promastigote culture. Infections were followed weekly by footpad measurement, and animals were culled after 10 weeks using approved schedule 1 methods before removal of footpad lesions. From each lesion, qPCR using fluorescence Sybr Green (Thermo Fisher) was performed in triplicate. DNA was extracted from the footpad lesion using an overnight incubation of proteinase K and use of the DNAeasy blood and tissue kit (Qiagen). DNA was eluted in 20 μL using the supplied buffer and quantified using a nanodrop. qPCR was carried out using two primer sets, one for the *Leishmania* kinetoplast DNA minicircle (Forward: AATGCGAGTGTTGCCCTTTTG, and Reverse: GCCGAACAACGCCATATTAACC) and the other for *Mus musculus* 5.8S RNA (Forward: CTCTTAGCGGTGGATCACTC, and Reverse: GTCGATGATCAATGTGTCCTGC). *Leishmania* minicircle cycles to threshold (Ct) values were normalised using the mouse Ct values.

### Statistical analysis

Statistical analyses were performed using Student’s *t*-test. A *p* value < 0.05 was considered statistically significant. Graphical illustrations and statistical analyses were performed using Prism software (GraphPad Software, Version 5).

## Supporting information

S1 Fig (A) Sequence alignment of *T*. *brucei* FAZ7, *Leishmania* FAZ7A (LmxM.19.0680) and FAZ7B (LmxM.19.0690). Sequences were aligned using MultAlin and the Fig was made using Espript (http://espript.ibcp.fr). Amino acids conserved are shaded in black. Dashes indicate gaps. Aminoacid (aa) positions of the kinesin motor domain using SMART prediction tool: 199aa to 564aa (*T*. *brucei* FAZ7), 80aa to 560aa (*Leishmania* FAZ7A) and 14aa to 502aa (*Leishmania* FAZ7B).
(TIF)

S2 Fig (A) Fluorescence micrographs of *L*. *mexicana* promastigotes expressing FAZ7A-mNG (green) and centrin4-mCh. (red). (B-C) Immunofluorescence assay on extracted cytoskeletons of *L*. *mexicana* promastigotes expressing FAZ7B-3xHA (red) and the fusion proteins of the FAZ cell body domain components FAZ2 and FAZ8 (green). DNA was stained with DAPI (blue). Scale bar: 5 μm. (D-E) Analysis of co-localization between the mNG-FAZ1 (green) and FAZ7B-mCh (red) signals in 3D-SIM (see S3 and S4 Movies); colocalization was measured in the mid-z-section of each of the images and calculated using the Pearson coefficient and van Steensel randomization method. The overall low values and the deep point at 0 (when the channels are not shifted) demonstrate exclusion of the two labeled proteins. This exclusion is also clearly visible in the 3D renderings of the images.
(TIF)

S3 Fig Diagnostic PCRs for verifying the correct integration of the tag/drug resistance markers and the absence of target gene wild-type allele(s). (A-E) Upper panels: schematic representation of each gene locus and primers (black arrows) used to confirm integration of the different drug resistant markers and loss of wild-type allele in the respective cell lines. Blasticidin (BLASTI), Phleomycin (PHLEO), Puromycin (PURO) and Neomycin (NEO). (A) PCR of parental cells expressing FAZ7B-3xHA. Lower panel: PCRs with gDNA from parental (1) and parental-FAZ7B-3xHA (2) using the indicated primers. (B) PCRs of FAZ7A, (C) FAZ7B and (D) FAZ7A+B null mutants. Lower panels: PCR amplification of gDNA from parental (P) and FAZ7 KO (KO) using the indicated primers. (E) PCR on parental and FAZ7B null mutant cells expressing FPC4-3xHA. PCR amplification of gDNA from parental (1), parental-FPC4-3xHA (2) and FAZ7B KO-FPC4-3xHA (3) using the indicated primers.
(TIF)

S4 Fig Growth curves and flagellum length measurement of FAZ7A and FAZ7A+B null mutants. Growth curves of (A) promastigotes and (B) axenic amastigotes of the parental, FAZ7A KO and FAZ7A+B double KO cell lines over a 4 days course. Cell density was determined by counting at 24 h intervals and mean ± SD of triplicate values was plotted. (C) Immunofluorescence labelling of parental, FAZ7A KO and FAZ7A+B double KO cell lines using anti-PFR2 antibody (green). DNA was stained with DAPI (blue). Scale bar: 5μm. (D) Flagellum length measurement of parental, FAZ7A KO and FAZ7A+B double KO cell lines. Cells were fixed with PFA at a density of 1x10^7^ cells/mL. (n = 300). Boxes and error bars indicate the median, upper and lower quartiles and 95th percentiles. *** p < 0.001 (Student’s *t*-test).
(TIF)

S5 Fig **(A-B) Basal body labeling in the FAZ7A null mutant**. Immunofluorescence labelling of extracted cytoskeleton of promastigotes from parental and FAZ7A KO cell lines. (A) YL1/2 antibody labelling (green). (B) Cells expressing centrin4-mNG (green). **(C) Subcellular localization of the basal body FAZ7A protein in the FAZ7B null mutant**. Immunofluorescence labelling of extracted cytoskeleton of promastigotes from parental and FAZ7B KO cell lines expressing FAZ7A-3xHA (green). DNA was labelled with DAPI (blue). Scale bar: 5μm.
(TIF)

S6 Fig Cytokinesis defects of FAZ7B null mutant promastigotes observed during time-lapse imaging. (A) Brightfield images representative of the four main types of cytokinesis defects observed. From left to right: cell division yielding two cells, plus one cytoplast devoid of flagellum; incomplete cytokinesis yielding sister cells remaining attached; incomplete cytokinesis yielding multiple rounds of division and multiple cytoplasts; ’catastrophic’ cell division event producing monster cells with multiple flagella and vesiculation of the cell body. (B) Distribution of the cytokinesis defect categories observed. See also S5–S7 Movies.
(TIF)

S7 Fig Ultrastructural analysis of promastigotes and amastigotes of the parental and FAZ7B null mutant. **(A)** Representative longitudinal sections of the flagellar pocket (FP) of the FAZ7B null mutant and parental cell lines are shown, both in promastigotes and axenic amastigotes. The FP overlay, with the bulbous lumen and the neck region, is overall preserved in promastigote and amastigote cells. Electron-dense areas, as well as attachment areas, around the FP neck are clearly visible. **(B)** Anarchic cell divisions in axenic amastigotes of the FAZ7 null mutant lead to ’monster’ cells. (a-b) A cell showing several kinetoplasts and flagellar pockets that appear randomly distributed throughout the cytoplasm. (c-d) A cell harbouring two pairs of nuclei showing two different chromatin patterns. Four transversal sections of FPs can be seen, one containing two flagella. The MTQ is visible close to two FPs (asterisks). N: nucleus; K: kinetoplast; F: flagellum; Scale bar: 1 μm.
(TIF)

S8 Fig Deletion of FAZ7B results in delayed incorporation of the membrane marker FM4-64FX into the flagellar pocket and altered flagellar pocket segregation. (A) Microphotographs representative of the flagellar pocket labelling with FM4-64FX (red) in the parental and FAZ7B KO cell lines. DNA was stained with DAPI (blue). Scale bar: 5 μm. (B) Quantification of FM4-64FX labelling in the flagellar pocket in 1N1K parental and FAZ7B KO cell lines; mean values ± SD of three independent experiments. ** p < 0.01 (Student’s *t*-test). (C) Microphotographs of representative parental and FAZ7B-KO promastigotes labelled with the flagellar pocket marker LmxM.23.0630 (SEC10) tagged with mCh (red) at late stage of cell division (2N2K2F). DNA was stained with DAPI (blue). Scale bar: 5 μm. (D) Quantification of divided FPs in late stage of cell division (2N2K2F) in parental and FAZ7B-KO cells expressing the FP marker LmxM.23.0630-mCh; mean values ± SD of three independent experiments. **p < 0.01 (Student’s *t*-test).
(TIF)

S9 Fig Subcellular localization of FAZ components in the FAZ7B null mutant. (A-H) Immunofluorescence labelling of whole cells from parental and FAZ7B null mutant cells expressing the indicated fusion proteins of FAZ components using anti-mNG (green). Only FAZ5 and, more evidently, ClpGM6 showed a change in localization. DNA was labelled with DAPI (blue). Scale bar: 5μm.
(TIF)

S10 Fig (A) Subcellular localization of the MTQ Spef1 protein remains unaltered in the FAZ7B null mutant. Immunofluorescence labelling of parental and FAZ7B KO cells expressing Spef1-mNG and FAZ7B-3xHA using anti-mNG (green) and anti-HA (red). DNA was labelled with DAPI (blue). Scale bar: 5μm.
(TIF)

S11 Fig Immunofluorescence labelling of promastigotes from parental and FAZ7B KO cell lines expressing mNG-BILBO1 and FPC4-mNG. Full view of microscopic fields. Both proteins displayed disorganized signals in the KO cells as compared with parental. Scale bar: 20μm.
(TIF)

S1 Movie Fly-through of image planes from z-stack images of the immunofluorescence labelling of promastigote cells expressing FAZ7B-3xHA (green) and SMP1-mCh (red). DNA was labelled with DAPI (blue).
(AVI)

S2 Movie Fly-through of image planes from z-stack images of the immunofluorescence labelling of promastigote cells expressing FAZ7B-3xHA (red) and mNG-FAZ1 (green). DNA was labelled with DAPI (blue).
(AVI)

S3 Movie 3D-SIM super resolution imaging. A 3D view of the respective subcellular localization of FAZ7B-mCherry (red) and mNG-FAZ1 (green) in a promastigote whole cell. Scale bar 1μm.
(AVI)

S4 Movie 3D-SIM super resolution imaging. A 3D view of the respective subcellular localization of FAZ7B-mCherry (red) and mNG-FAZ1 (green) in a promastigote extracted cytoskeleton. Scale bar 1μm.
(AVI)

S5 Movie Abnormal cell division event in the promastigote FAZ7B null mutant cell line filmed in time lapse microscopy over 11 hrs. This cell division event is near normal: two cells and a small cytoplast are produced; one of the resulting cells eventually divides.
(AVI)

S6 Movie Abnormal cell division event in the promastigote FAZ7B null mutant cell line filmed in time lapse microscopy over 12 hrs. In this event, cytokinesis is incomplete, yielding two and then four cells remaining attached, even after 8 hrs following the cell division start; in the same conditions, parental cells took about 1–1.5 hr to complete cytokinesis, from the emergence of the second flagellum to complete cell separation.
(AVI)

S7 Movie Abnormal cell division event in the promastigote FAZ7B null mutant cell line filmed in time lapse microscopy over 12 hrs. Two neighbouring cells start dividing simultaneously. A series of incomplete cell division rounds leads to the production of unseparated cells and cytoplasts, and finally to a ’monster’ cell with multiple cell bodies, flagella and probably nuclei.
(AVI)

S8 Movie Swimming tracks from video microscopy of the parental cell line. (AVI)

S9 Movie Swimming tracks from video microscopy of the FAZ7B null mutant. (AVI)

S1 Table Primers used in this study. (DOC)
