# Supplementary figures and images for: The kinesin of the flagellum attachment zone in Leishmania is required for cell morphogenesis, cell division and virulence in the mammalian host
Source: PLoS Pathog. 2021 Jun 18;17(6):e1009666. doi: 10.1371/journal.ppat.1009666 (PMC8244899; doi:10.1371/journal.ppat.1009666)

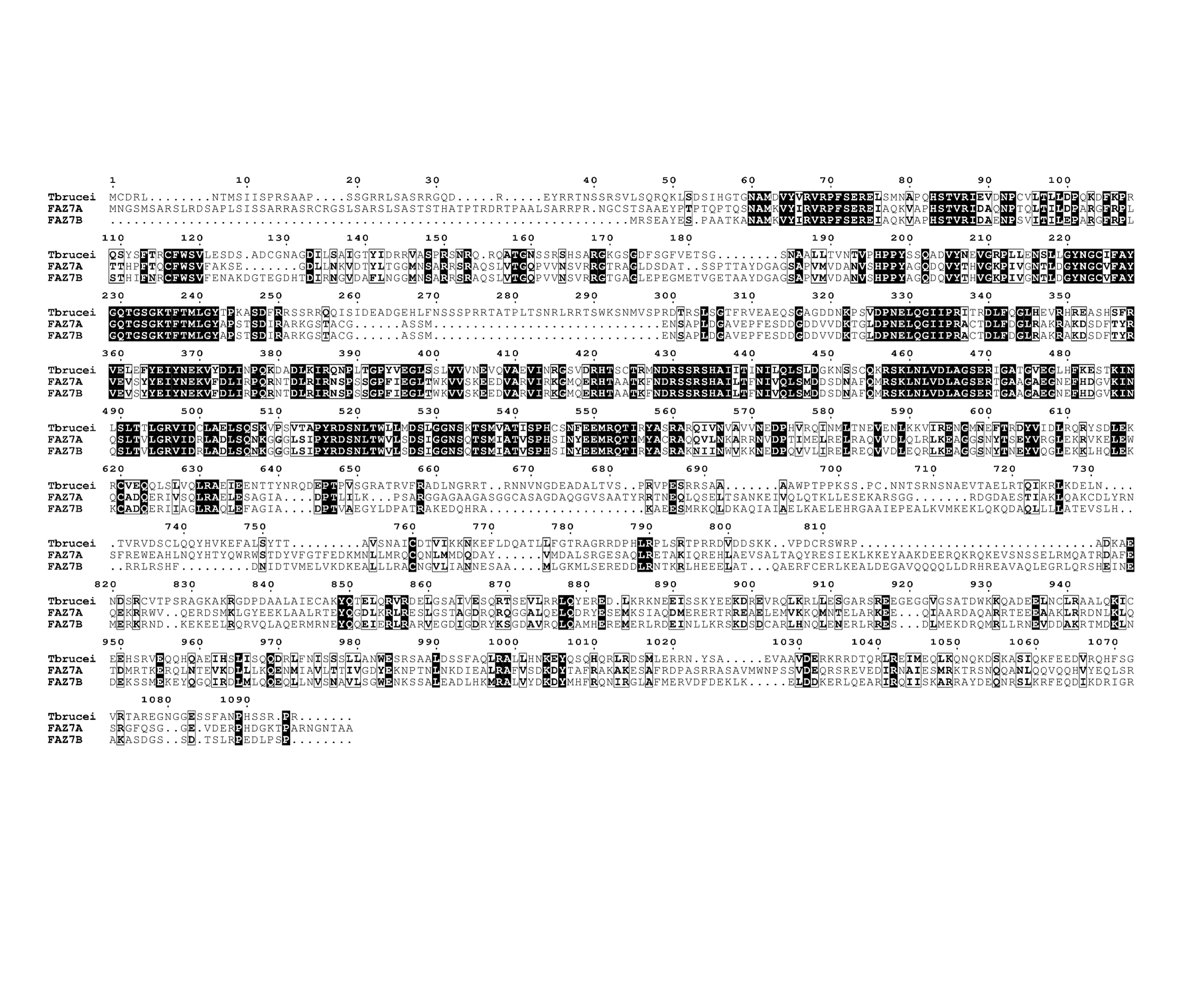

Supplement: S1 Fig — (A) Sequence alignment of T. brucei FAZ7, Leishmania FAZ7A (LmxM.19.0680) and FAZ7B (LmxM.19.0690). Sequences were aligned using MultAlin and the Fig was made using Espript (http://espript.ibcp.fr). Amino acids conserved are shaded in black. Dashes indicate gaps. Aminoacid (aa) positions of the kinesin motor domain using SMART prediction tool: 199aa to 564aa (T. brucei FAZ7), 80aa to 560aa (Leishmania FAZ7A) and 14aa to 502aa (Leishmania FAZ7B). (TIF) [file ppat.1009666.s001.tif]

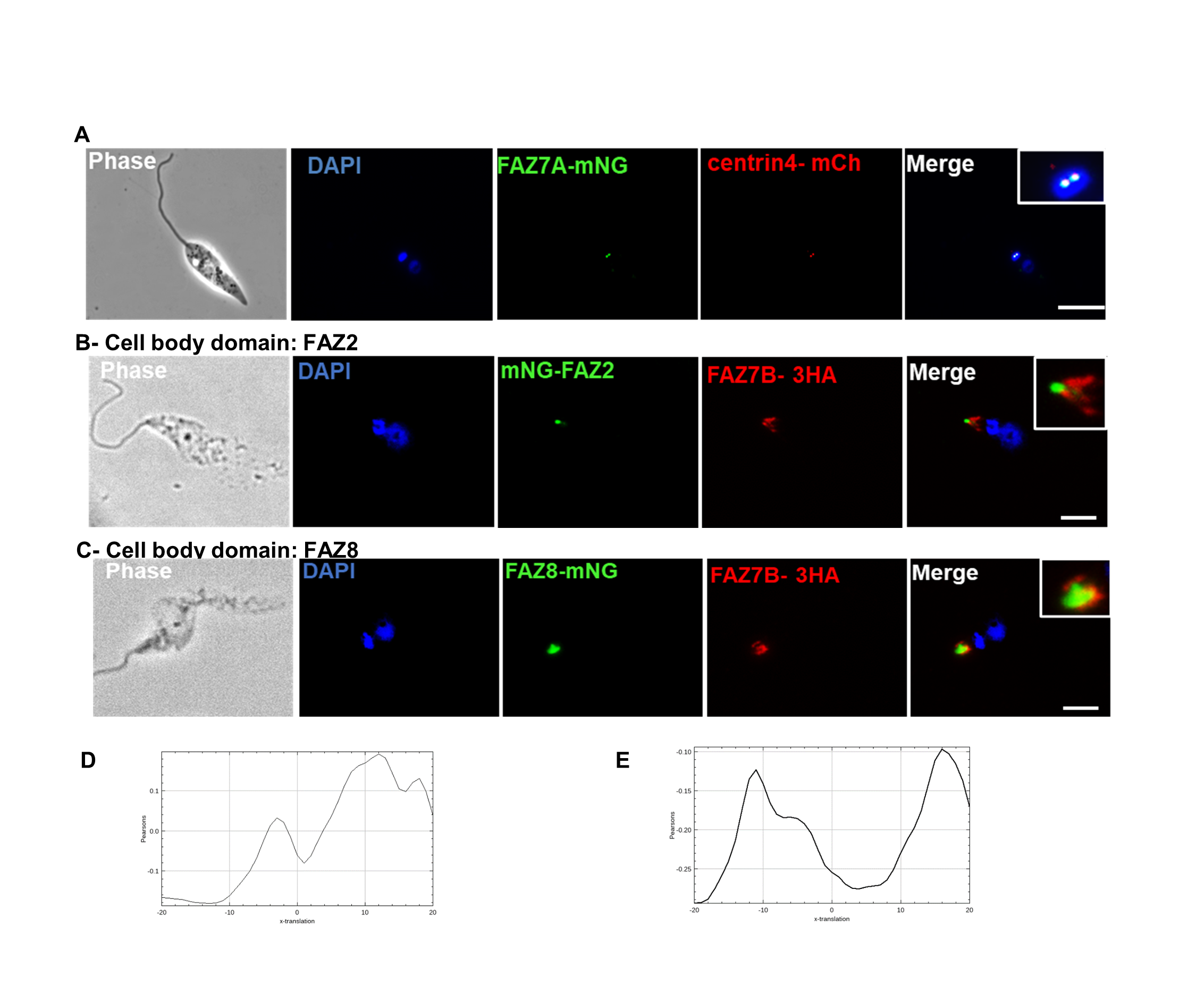

Supplement: S2 Fig — (A) Fluorescence micrographs of L. mexicana promastigotes expressing FAZ7A-mNG (green) and centrin4-mCh. (red). (B-C) Immunofluorescence assay on extracted cytoskeletons of L. mexicana promastigotes expressing FAZ7B-3xHA (red) and the fusion proteins of the FAZ cell body domain components FAZ2 and FAZ8 (green). DNA was stained with DAPI (blue). Scale bar: 5 μm. (D-E) Analysis of co-localization between the mNG-FAZ1 (green) and FAZ7B-mCh (red) signals in 3D-SIM (see S3 and S4 Movies); colocalization was measured in the mid-z-section of each of the images and calculated using the Pearson coefficient and van Steensel randomization method. The overall low values and the deep point at 0 (when the channels are not shifted) demonstrate exclusion of the two labeled proteins. This exclusion is also clearly visible in the 3D renderings of the images. (TIF) [file ppat.1009666.s002.tif]

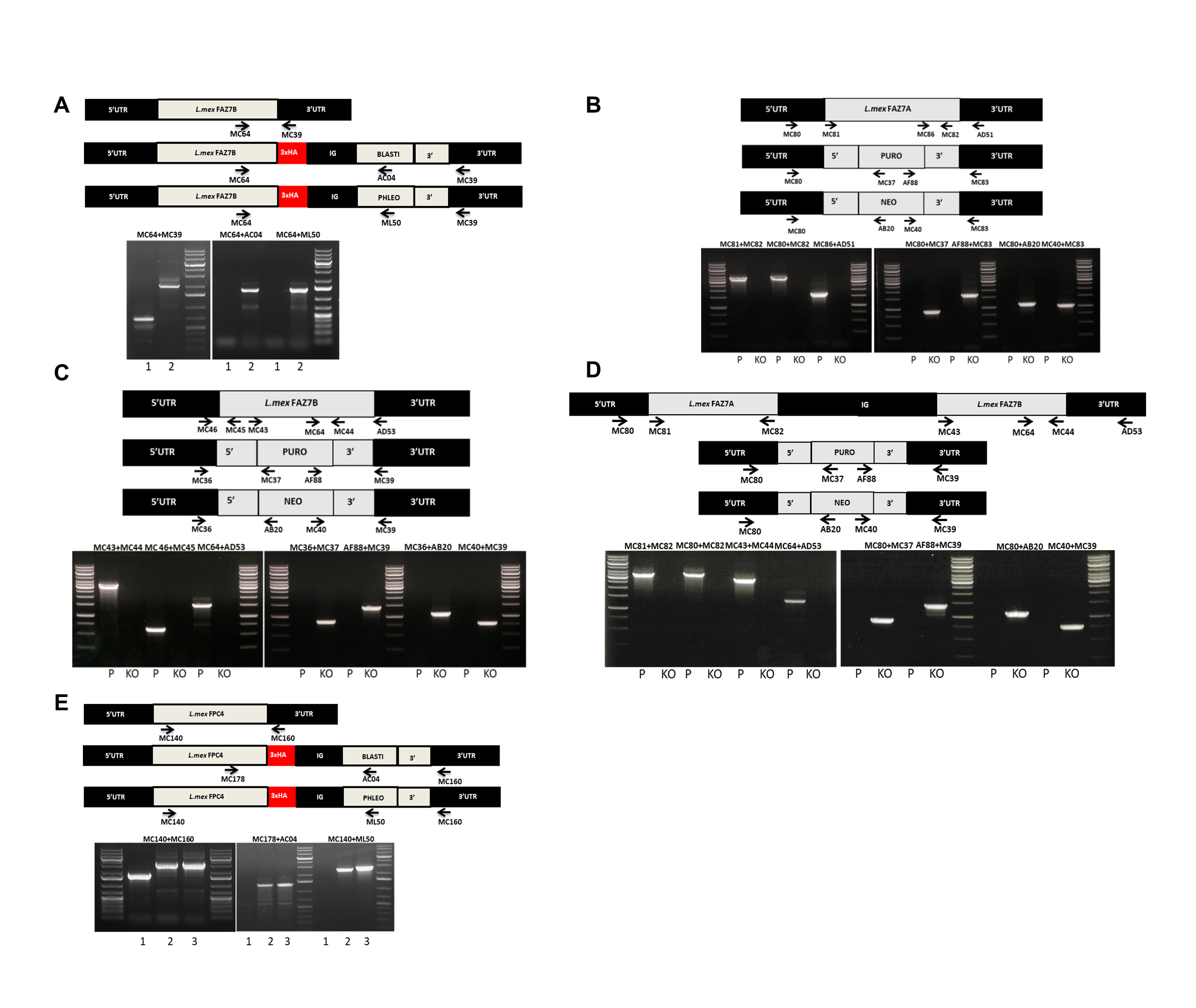

Supplement: S3 Fig — (A-E) Upper panels: schematic representation of each gene locus and primers (black arrows) used to confirm integration of the different drug resistant markers and loss of wild-type allele in the respective cell lines. Blasticidin (BLASTI), Phleomycin (PHLEO), Puromycin (PURO) and Neomycin (NEO). (A) PCR of parental cells expressing FAZ7B-3xHA. Lower panel: PCRs with gDNA from parental (1) and parental-FAZ7B-3xHA (2) using the indicated primers. (B) PCRs of FAZ7A, (C) FAZ7B and (D) FAZ7A+B null mutants. Lower panels: PCR amplification of gDNA from parental (P) and FAZ7 KO (KO) using the indicated primers. (E) PCR on parental and FAZ7B null mutant cells expressing FPC4-3xHA. PCR amplification of gDNA from parental (1), parental-FPC4-3xHA (2) and FAZ7B KO-FPC4-3xHA (3) using the indicated primers. (TIF) [file ppat.1009666.s003.tif]

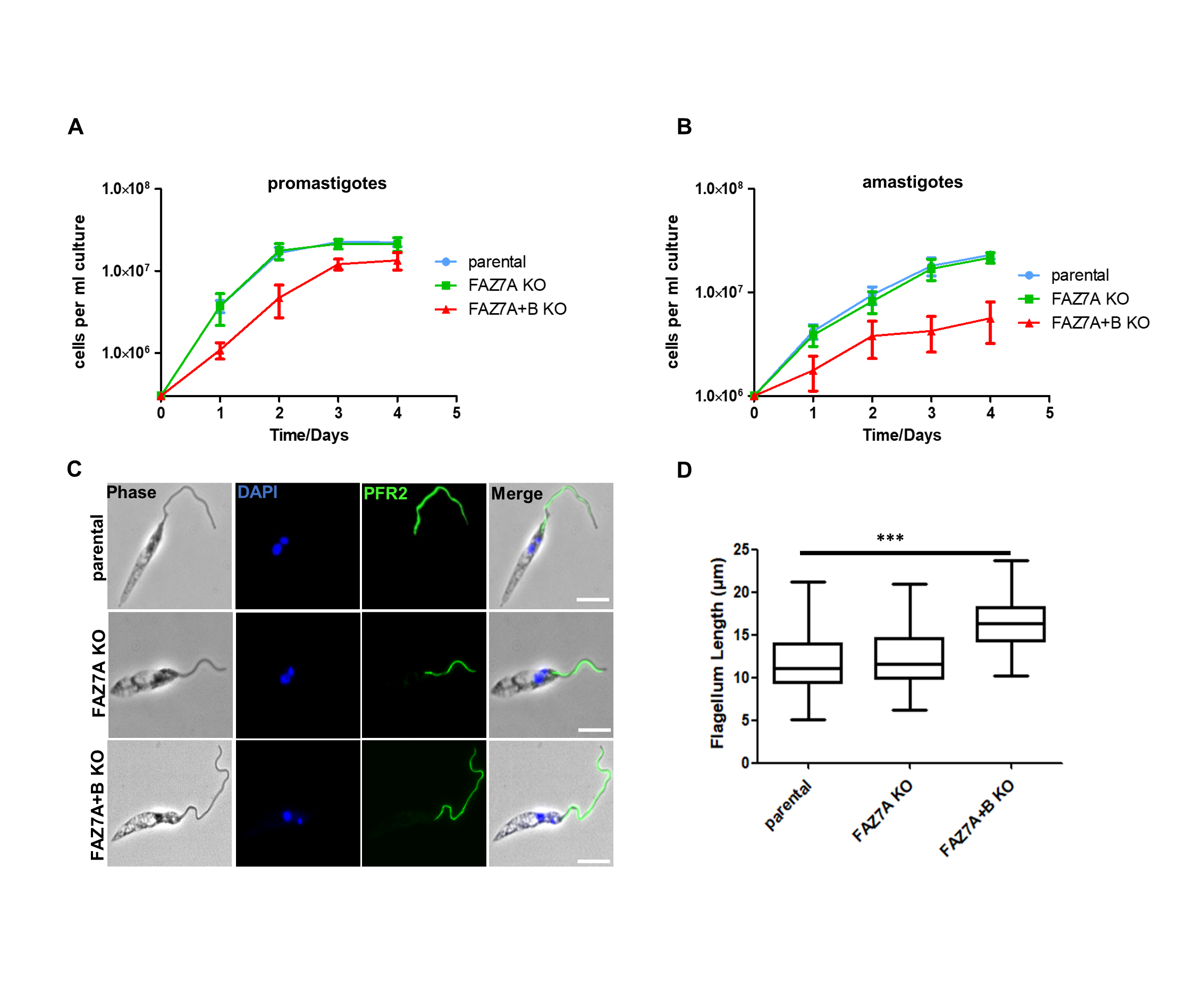

Supplement: S4 Fig — Growth curves of (A) promastigotes and (B) axenic amastigotes of the parental, FAZ7A KO and FAZ7A+B double KO cell lines over a 4 days course. Cell density was determined by counting at 24 h intervals and mean ± SD of triplicate values was plotted. (C) Immunofluorescence labelling of parental, FAZ7A KO and FAZ7A+B double KO cell lines using anti-PFR2 antibody (green). DNA was stained with DAPI (blue). Scale bar: 5μm. (D) Flagellum length measurement of parental, FAZ7A KO and FAZ7A+B double KO cell lines. Cells were fixed with PFA at a density of 1x10^7^ cells/mL. (n = 300). Boxes and error bars indicate the median, upper and lower quartiles and 95th percentiles. *** p < 0.001 (Student’s t-test). (TIF) [file ppat.1009666.s004.tif]

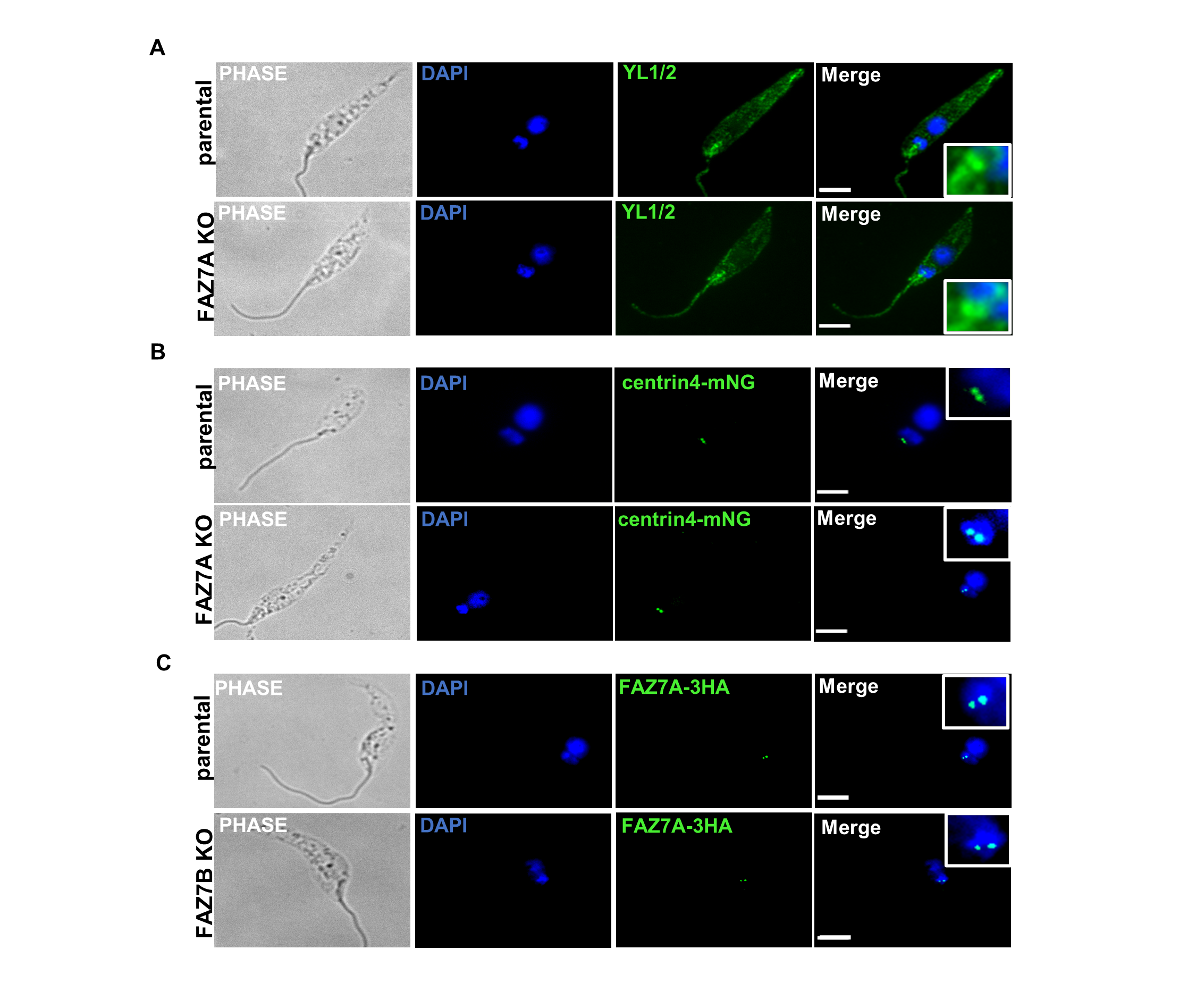

Supplement: S5 Fig — (A-B) Basal body labeling in the FAZ7A null mutant. Immunofluorescence labelling of extracted cytoskeleton of promastigotes from parental and FAZ7A KO cell lines. (A) YL1/2 antibody labelling (green). (B) Cells expressing centrin4-mNG (green). (C) Subcellular localization of the basal body FAZ7A protein in the FAZ7B null mutant. Immunofluorescence labelling of extracted cytoskeleton of promastigotes from parental and FAZ7B KO cell lines expressing FAZ7A-3xHA (green). DNA was labelled with DAPI (blue). Scale bar: 5μm. (TIF) [file ppat.1009666.s005.tif]

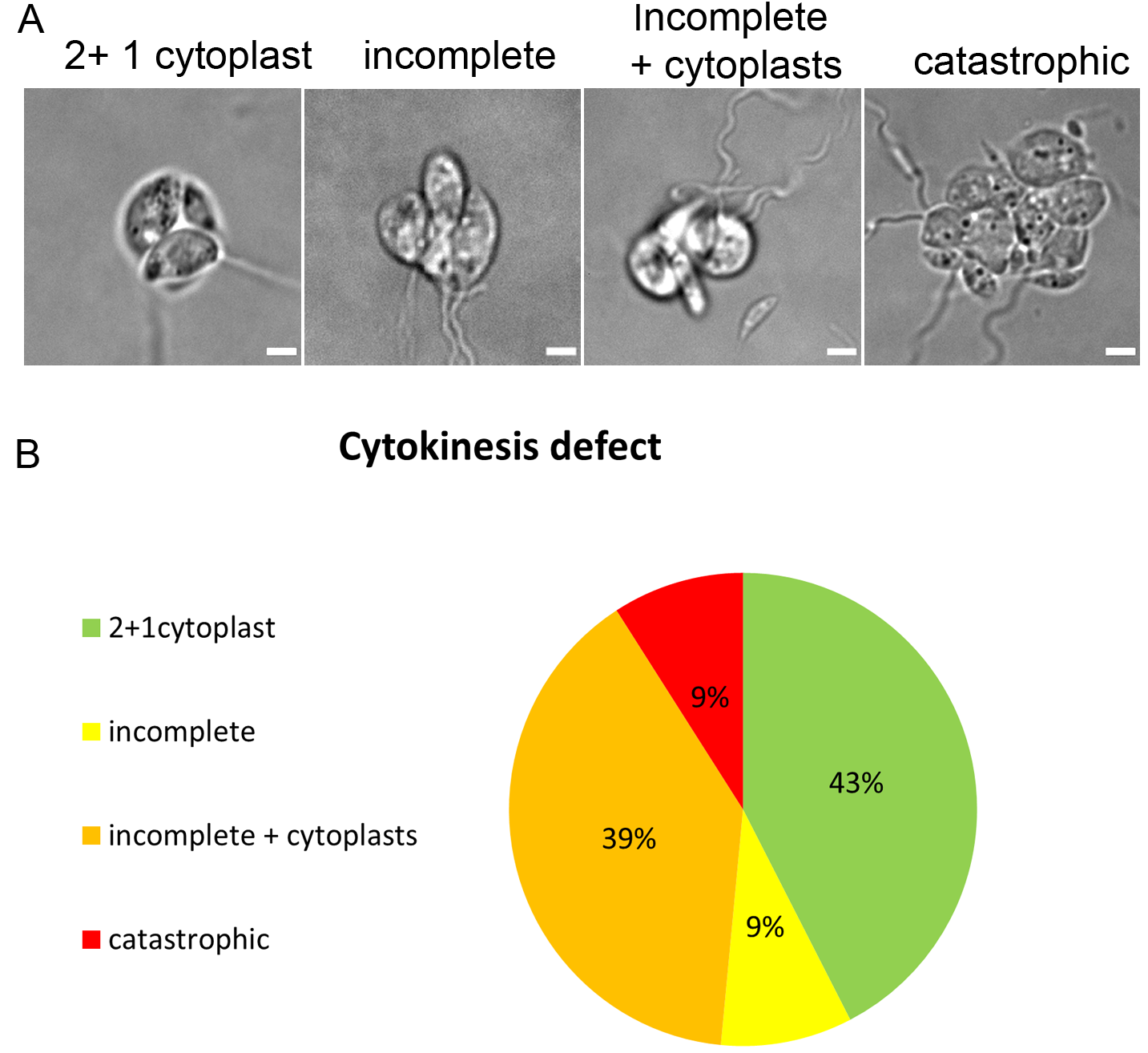

Supplement: S6 Fig — (A) Brightfield images representative of the four main types of cytokinesis defects observed. From left to right: cell division yielding two cells, plus one cytoplast devoid of flagellum; incomplete cytokinesis yielding sister cells remaining attached; incomplete cytokinesis yielding multiple rounds of division and multiple cytoplasts; ’catastrophic’ cell division event producing monster cells with multiple flagella and vesiculation of the cell body. (B) Distribution of the cytokinesis defect categories observed. See also S5–S7 Movies. (TIF) [file ppat.1009666.s006.tif]

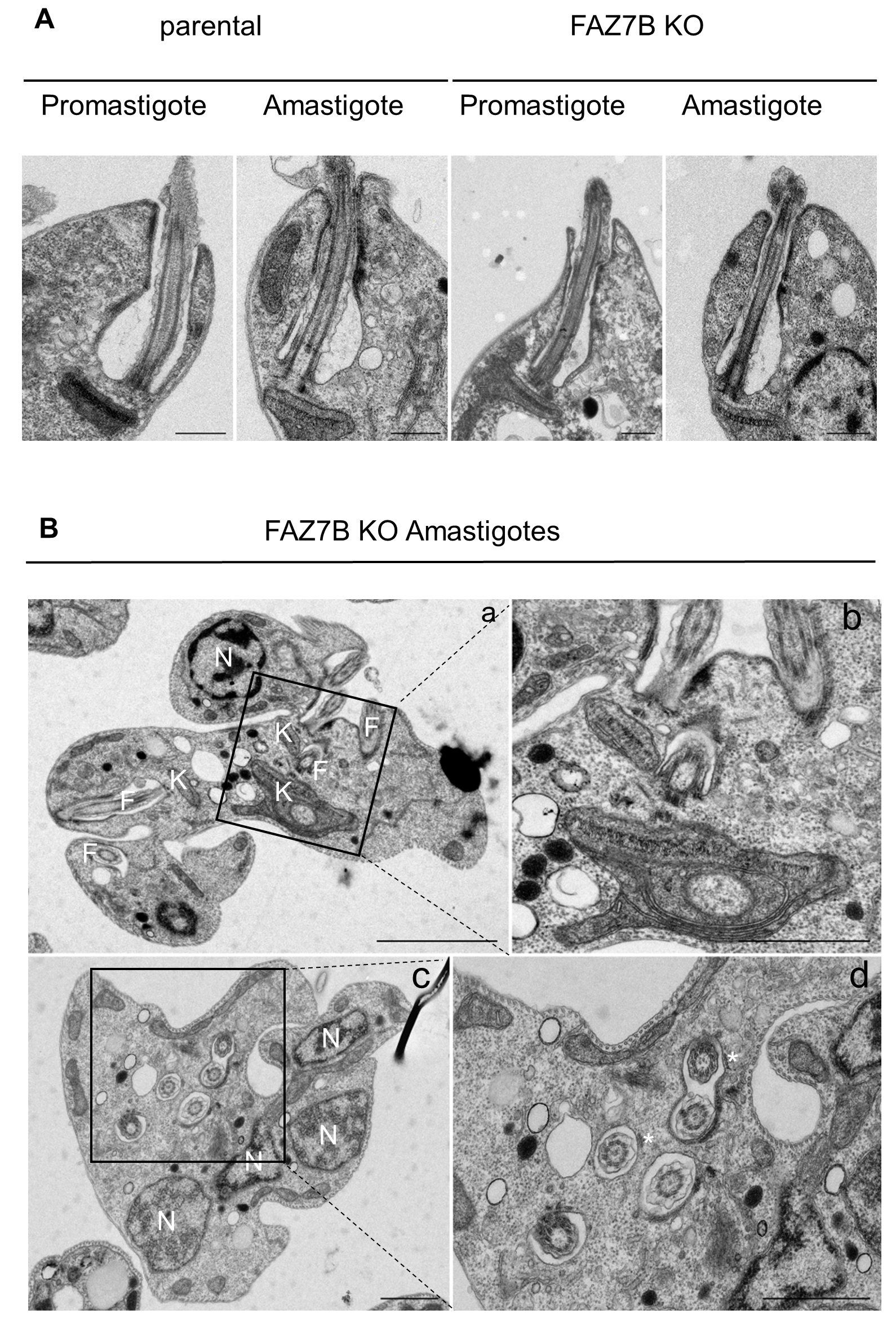

Supplement: S7 Fig — (A) Representative longitudinal sections of the flagellar pocket (FP) of the FAZ7B null mutant and parental cell lines are shown, both in promastigotes and axenic amastigotes. The FP overlay, with the bulbous lumen and the neck region, is overall preserved in promastigote and amastigote cells. Electron-dense areas, as well as attachment areas, around the FP neck are clearly visible. (B) Anarchic cell divisions in axenic amastigotes of the FAZ7 null mutant lead to ’monster’ cells. (a-b) A cell showing several kinetoplasts and flagellar pockets that appear randomly distributed throughout the cytoplasm. (c-d) A cell harbouring two pairs of nuclei showing two different chromatin patterns. Four transversal sections of FPs can be seen, one containing two flagella. The MTQ is visible close to two FPs (asterisks). N: nucleus; K: kinetoplast; F: flagellum; Scale bar: 1 μm. (TIF) [file ppat.1009666.s007.tif]

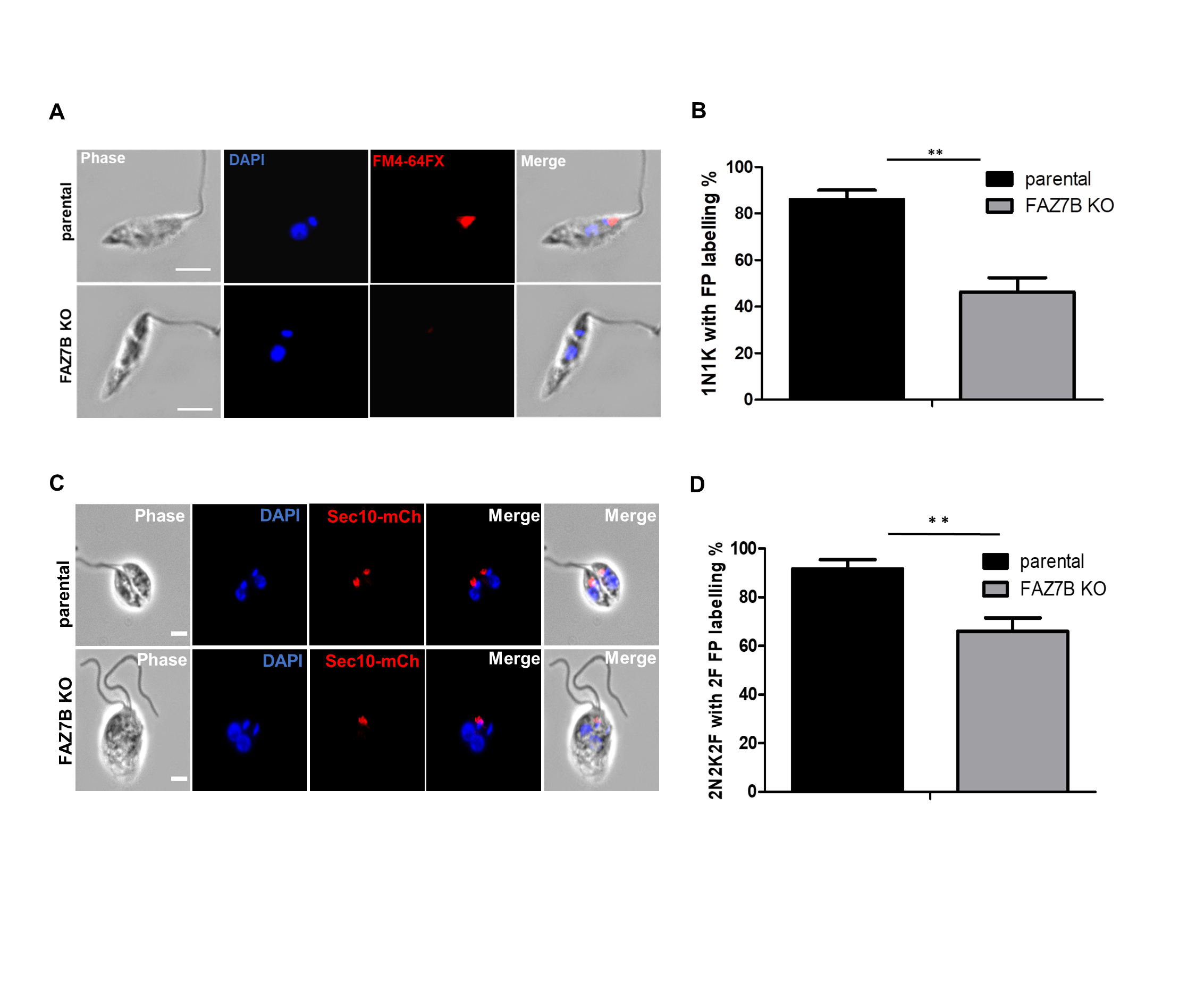

Supplement: S8 Fig — (A) Microphotographs representative of the flagellar pocket labelling with FM4-64FX (red) in the parental and FAZ7B KO cell lines. DNA was stained with DAPI (blue). Scale bar: 5 μm. (B) Quantification of FM4-64FX labelling in the flagellar pocket in 1N1K parental and FAZ7B KO cell lines; mean values ± SD of three independent experiments. ** p < 0.01 (Student’s t-test). (C) Microphotographs of representative parental and FAZ7B-KO promastigotes labelled with the flagellar pocket marker LmxM.23.0630 (SEC10) tagged with mCh (red) at late stage of cell division (2N2K2F). DNA was stained with DAPI (blue). Scale bar: 5 μm. (D) Quantification of divided FPs in late stage of cell division (2N2K2F) in parental and FAZ7B-KO cells expressing the FP marker LmxM.23.0630-mCh; mean values ± SD of three independent experiments. **p < 0.01 (Student’s t-test). (TIF) [file ppat.1009666.s008.tif]

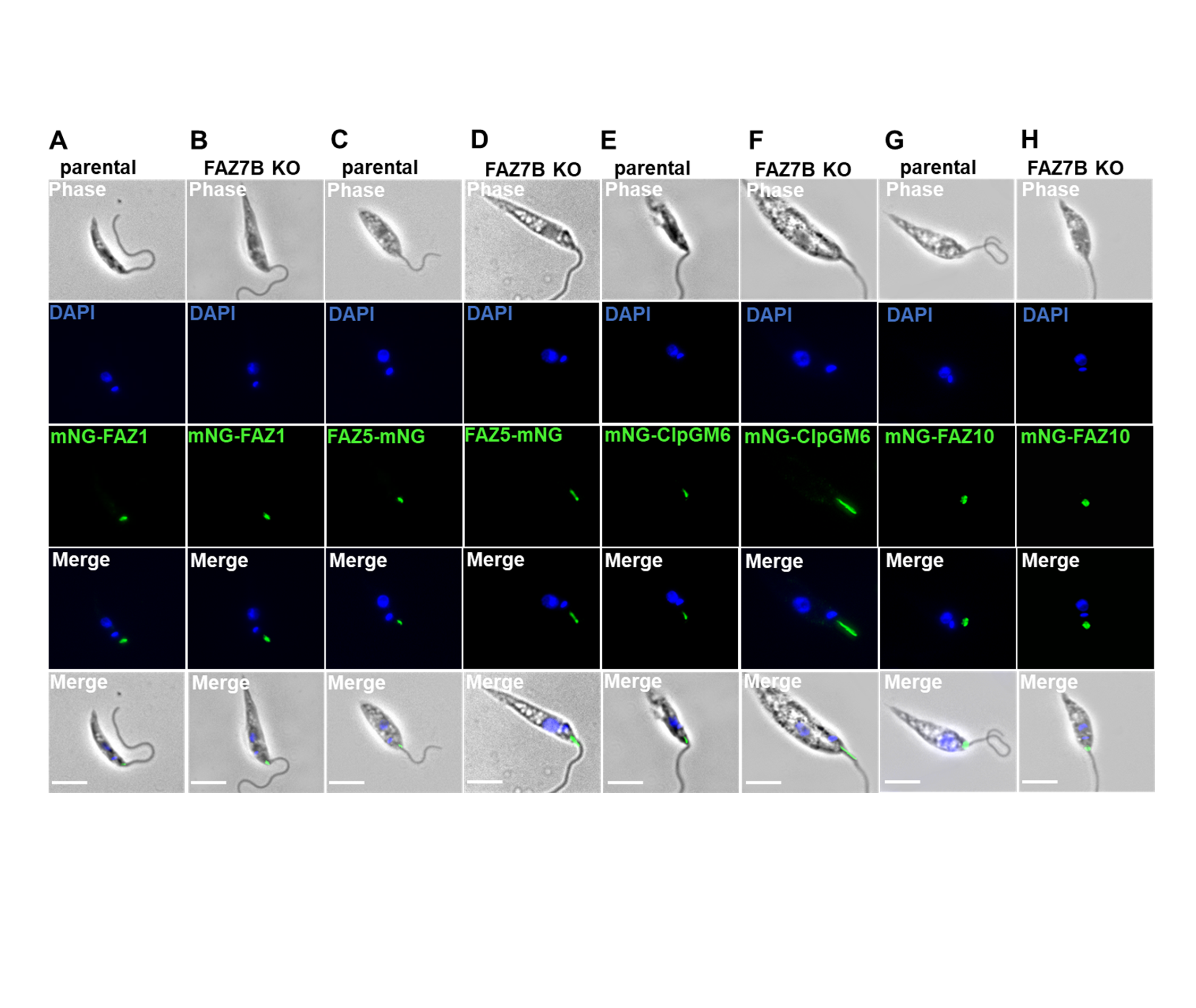

Supplement: S9 Fig — (A-H) Immunofluorescence labelling of whole cells from parental and FAZ7B null mutant cells expressing the indicated fusion proteins of FAZ components using anti-mNG (green). Only FAZ5 and, more evidently, ClpGM6 showed a change in localization. DNA was labelled with DAPI (blue). Scale bar: 5μm. (TIF) [file ppat.1009666.s009.tif]

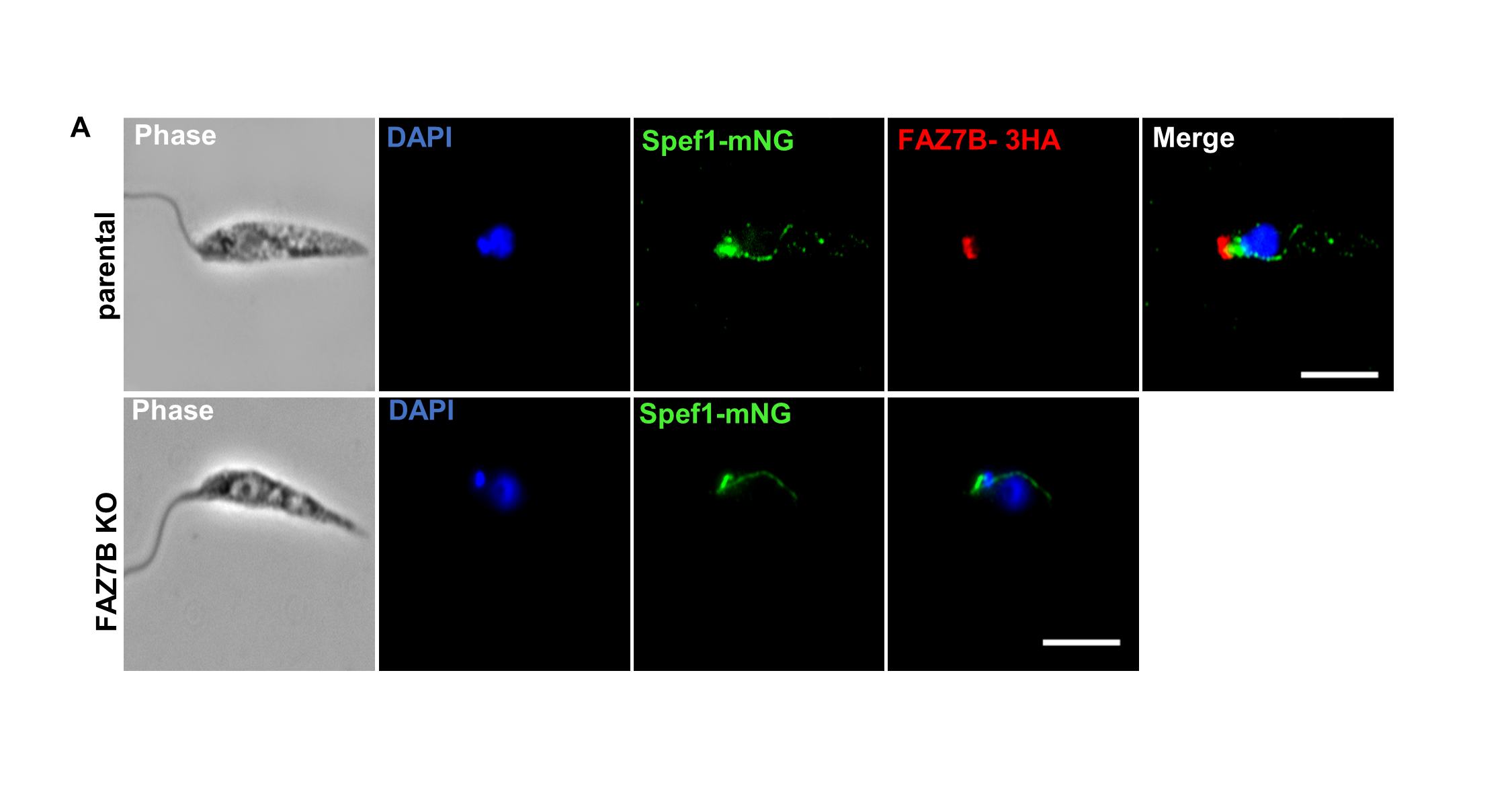

Supplement: S10 Fig — (A) Subcellular localization of the MTQ Spef1 protein remains unaltered in the FAZ7B null mutant. Immunofluorescence labelling of parental and FAZ7B KO cells expressing Spef1-mNG and FAZ7B-3xHA using anti-mNG (green) and anti-HA (red). DNA was labelled with DAPI (blue). Scale bar: 5μm. (TIF) [file ppat.1009666.s010.tif]

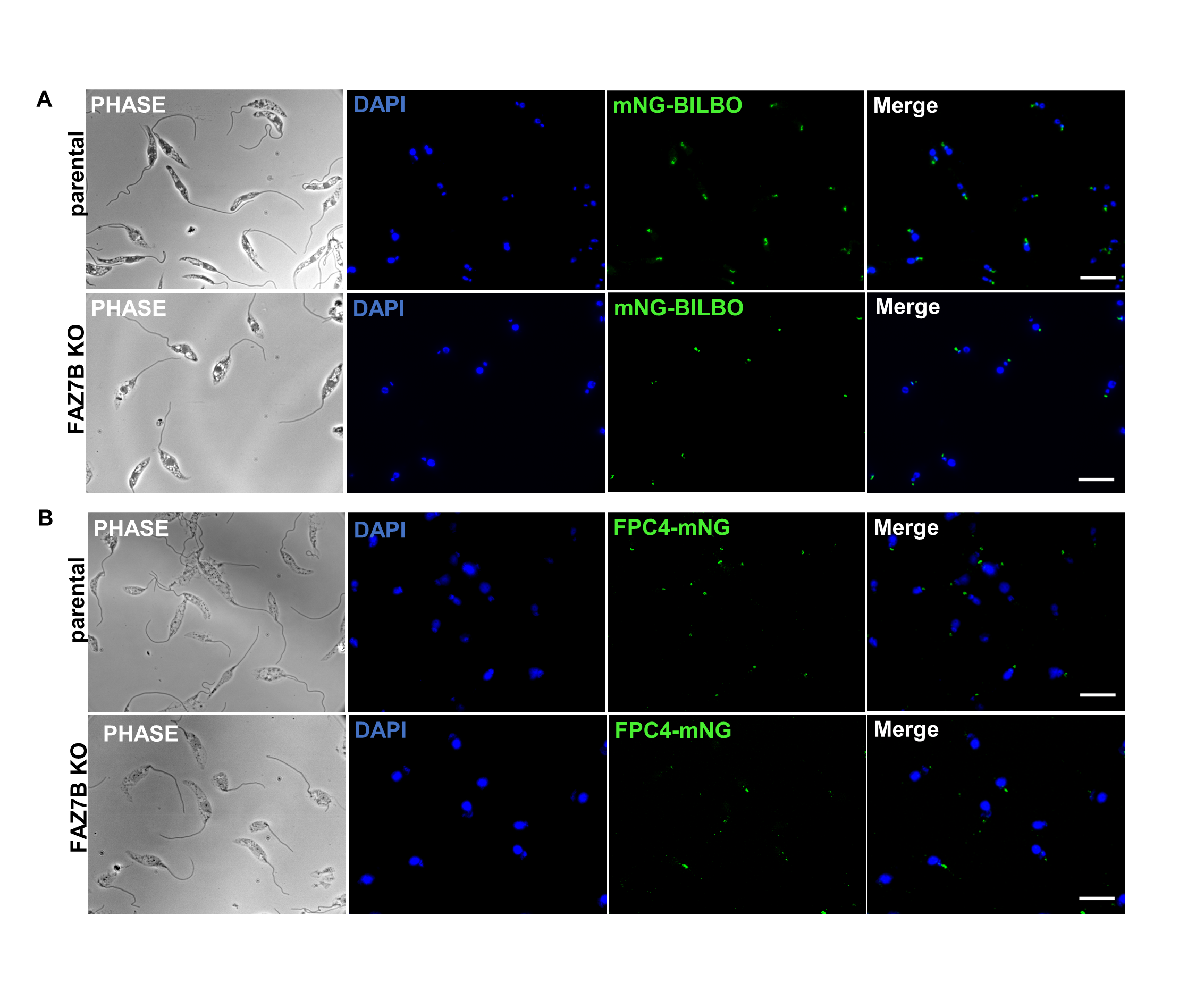

Supplement: S11 Fig — Full view of microscopic fields. Both proteins displayed disorganized signals in the KO cells as compared with parental. Scale bar: 20μm. (TIF) [file ppat.1009666.s011.tif]
