# Supplementary material for: The kinesin of the flagellum attachment zone in Leishmania is required for cell morphogenesis, cell division and virulence in the mammalian host
Source: PLoS Pathog. 2021 Jun 18;17(6):e1009666. doi: 10.1371/journal.ppat.1009666 (PMC8244899; doi:10.1371/journal.ppat.1009666)
Supplement: S1 Table — (DOC) [file ppat.1009666.s021.doc]

**S1 Table.** Primers used in this study.

| **Primers** | **5' to 3' Sequence** | **Target** |
| --- | --- | --- |
| MC64 dir | GTGCTCGTCTGCATAAT | FAZ7B ORF |
| MC39 rev | CGAGCTAAGACGGAGAGGGG | FAZ7B 3’UTR |
| AC04 rev | tgctctttcaatgagggtgg | blasticidin |
| ML50 rev | GGAACGGCACTGGTCAACTT | Phleomycin |
| MC80 dir | CAGCGTGCCGCCTGAGTACTC | FAZ7A 5’UTR |
| MC81 dir | ATGAACGGATCCATGTCTGC | FAZ7A ORF |
| MC82 rev | GCA GGG GTC TTG CCG TCA TG | FAZ7A ORF |
| MC86 dir | GCGATAGCATGAAGCTTG | FAZ7A ORF |
| AD51 rev | GGTGCTTCACCACCATTGC | FAZ7A 3’UTR |
| MC37 rev | TCAATGTGTCGATCTGGGTCAAC | PUROMYCIN |
| AF88 dir | CCCAGATCGACACATTGAGCG | PUROMYCIN |
| MC83 rev | GGA CCC GTG ACC TAA CAT CC | FAZ7A 3’UTR |
| AB20 rev | ATCGACAAGACCGGCTTCC | nEOMYCIN |
| MC40 dir | ACCGCTTCCTCGTGCTTTA | NEOMYCIN |
| MC43 dir | GCGCAGTGAGGCTTATGAGTCTC | FAZ7B orf |
| MC44 rev | GCTACCATCGCTCGCCTTGGCACGG | FAZ7B orf |
| MC45 rev | GAGACTCATAAGCCTCACTGCGC | FAZ7B orf |
| MC46 dir | GCC CAA GGG AAA GAG CAC CC | faz7B 5’UTR |
| AD53 rev | AACAGCTTGTCTTTCTGAAAGGG | FAZ7B 3’UTR |
| MC36 dir | GTGTGTATGTGCGTGCATGC | FAZ7B 5’UTR |
| MC140 dir | CAGCTTTACGCGGTGACCG | FPC4 ORF |
| MC160 rev | CTGGCTGCAACGCCACCC | FPC4 3’UTR |
| MC178 dir | GTCGAAGTCGCGTGTGGTGG | FPC4 ORF |
| MC123 dir | CAAGCGAATTCCATATGCGCAGTGAGGCTTATGAG (NdeI) | FAZ7B ORF |
| MC125 rev | TCGGAGGAGGCCATGGTACCTGGGGAGGGGAGGTCTTC (KpnI) | FAZ7B ORF |
| MC126 dir | CAAGCGAATTCCATATGGATCCGCAGGTGGTACTC (NdeI) | FAZ7B ORF |
| MC141 dir | GTAGC**GCCGCG**ACCTTCACGATGCTGGGC | FAZ7B ORF |
| MC142 rev | AGGT**CGCGGC**GCTACCCGTCTGCCCGTAG | FAZ7B ORF |
| MC143 dir | GGCCAGTGCCAAGCTTGTTAACCCACGGTTGCCTC (HindIII, HpaI) | FAZ7B 5’UTR |
| MC144 rev | TCTTGTCGGTAAGCTTGGGAATTGACTTCGCGCGTG (HindIII) | FAZ7B 5’UTR |
| MC145 dir | GCAGGAAAGAACATGTCGTCTTAGCTCGCTCTTTTCCG (PciI) | FAZ7B 3’UTR |
| MC146 rev | CCTTTTGCTCACATGTCAATTGGGGACGGGACGGG (Pcil, MfeI) | FAZ7B 3’UTR |
| MC147 dir | gaaattaatacgactcactatagg***CTATACACGTCGCTTCGTTC***gttttagagctagaaatagc | 5’guide FAZ7B |
| MC148 dir | gaaattaatacgactcactatagg***GAGAGGGGTGTGGCGGCGAT***gttttagagctagaaatagc | 3’Guide FAZ7B |

^a^ Restriction sites are underlined and specified into brackets.

^b^ Mutagenized bases are shown in bold.

^c^ sgRNA target sites are indicated in bold italics
